# Marine-Derived Rare Actinomycetes: Metabolites and Their Biosynthesis

**DOI:** 10.3390/md24080284

**Published:** 2026-08-19

**Authors:** Juwan Son, Hyeon Seung Park, Sang Heon Jung, Min Seo Heo, Yun Kwon, Munhyung Bae

**Affiliations:** 1 College of Pharmacy, Gachon University, Incheon 21936, Republic of Korea; ths3120@gachon.ac.kr (J.S.); fly6778@gachon.ac.kr (S.H.J.); 2 Research Institute of Pharmaceutical Science, College of Pharmacy, Kyungpook National University, Daegu 41566, Republic of Korea; ss522s@knu.ac.kr (H.S.P.); iulike103@knu.ac.kr (M.S.H.)

**Keywords:** marine-derived rare actinomycetes, biosynthetic gene clusters, genome mining, polyketides, nonribosomal peptides, diketopiperazines, siderophores, tailoring enzymes, heterologous expression

## Abstract

Marine-derived rare actinomycetes are a chemically prolific yet underexploited source of structurally diverse secondary metabolites. In this review, rare actinomycetes are operationally defined as marine-derived non-*Streptomyces* actinomycetes that remain comparatively underexplored yet possess demonstrated or predicted capacity for specialized-metabolite biosynthesis. Genome sequencing has revealed that their biosynthetic potential greatly exceeds the range of metabolites recovered under standard cultivation conditions. However, many reported compounds remain only loosely associated with the gene clusters that encode them. This review provides a biosynthesis-centered perspective on marine-derived rare actinomycetes, focusing on secondary metabolites for which biosynthetic gene clusters (BGCs) or pathways have been proposed, experimentally assessed, or functionally validated. It focuses on compounds reported after 2017, along with earlier metabolites whose biosynthetic origins were resolved only later. Representative examples are organized by genus and structural class and weighed according to the level of evidence linking each metabolite to its BGC, ranging from bioinformatic prediction and metabolomic correlation to validation by gene inactivation, heterologous expression, and enzymatic characterization. The surveyed metabolites include polyketides, nonribosomal peptides, polyketide synthase-nonribosomal peptide synthetase (PKS-NRPS) hybrids, siderophores, angucyclines, anthracyclines, macrolides, diketopiperazine derivatives, and other unusual scaffolds. Collectively, these findings indicate how integrating genome mining, metabolomics, and molecular networking with targeted biosynthetic experiments can accelerate marine natural product discovery and unravel novel enzymatic functions and biosynthetic mechanisms in rare actinomycetes.

## 1. Introduction

Marine-derived actinomycetes are a well-established source of structurally diverse and biologically active secondary metabolites, including chemical scaffolds that remain uncommon among metabolites reported from terrestrial microorganisms [1,2]. Actinomycetes have played a central role in anti-infective drug discovery for much of the past century and have yielded numerous antibiotic classes that remain in clinical use [3,4]. More broadly, natural products and their derivatives continue to account for a substantial proportion of approved small-molecule therapeutics, underscoring the enduring value of microbial secondary metabolism as a source of pharmacologically relevant chemical matter [5]. Historically, microbial natural product discovery has been dominated by the genus *Streptomyces*. The increasing frequency of rediscovery within extensively investigated *Streptomyces* collections, however, has stimulated greater interest in non-*Streptomyces*, or “rare” actinomycetes as sources of less-explored chemical and biosynthetic diversity [1,2,3]. In this review, rare actinomycetes are operationally defined as marine-derived non-*Streptomyces* actinomycetes that remain comparatively understudied but possess demonstrated or predicted capacity to produce structurally unusual specialized metabolites. Their isolation and cultivation can nevertheless be challenging, which continues to constrain the taxonomic and chemical space accessible to experimental investigation [6].

Marine and marine-associated rare actinomycetes have been recovered from sediments, sponges, corals, algae, mangroves, and deep-sea environments [1,2]. These habitats expose microorganisms to distinctive combinations of salinity, hydrostatic pressure, temperature, nutrient limitation, and biotic interactions that can shape the maintenance and expression of specialized metabolic pathways [4,7]. The occurrence of obligately marine taxa such as *Salinispora*, together with the frequent recovery of rare actinomycetes from marine invertebrate-associated communities, has further emphasized the marine environment as a productive setting for specialized-metabolite discovery [4,7]. Chemically, these organisms produce polyketides, nonribosomal peptides, PKS–NRPS hybrids, siderophores, diketopiperazine derivatives, macrolides, angucyclines, anthracyclines, xanthones, and other biosynthetically unusual scaffolds. Their reported activities span antibacterial, antifungal, cytotoxic, anti-inflammatory, enzyme-inhibitory, and metal-binding functions [1,2]. The continuing contribution of marine microorganisms to newly reported natural products further illustrates the chemical productivity of this source [8].

Genome sequencing has substantially altered how this biosynthetic potential is interrogated. Comparative genomic studies consistently show that individual actinomycete genomes encode substantially more biosynthetic gene clusters (BGCs) than can be accounted for by metabolites detected under routine laboratory cultivation [9,10,11]. Many of these clusters are transcriptionally silent or weakly expressed, producing a persistent disparity between genetically encoded biosynthetic capacity and experimentally observed chemistry [10,12]. Genome mining therefore complements conventional bioactivity-guided isolation by allowing cryptic pathways to be recognized and prioritized before their products are known [11,13]. This transition has recast the BGC as a practical unit of natural product discovery and has been accelerated by computational approaches that identify, classify, and increasingly predict features of specialized-metabolite pathways from genomic and metagenomic sequence data [14,15].

As biosynthetic sequences have become increasingly accessible, however, the principal challenge has shifted. Detecting a candidate BGC is now often considerably easier than establishing the metabolite that it produces, determining whether the pathway is expressed, and defining the enzymatic transformations that generate the final chemical structure. This problem is particularly acute for marine-derived rare actinomycetes, many of which grow slowly, remain comparatively difficult to manipulate genetically, or produce target metabolites only under restricted cultivation conditions [13]. Consequently, sequence-based prediction alone provides only a hypothesis of biosynthetic origin; robust genotype–chemotype assignments increasingly require integration with metabolomic correlation, genetic interrogation, heterologous expression, and, ultimately, biochemical characterization of individual pathway steps [11,15].

Previous reviews have comprehensively described the isolation, taxonomy, metabolite diversity, and biological activities of marine-derived rare actinomycetes, particularly compounds reported through 2017 [1,2]. The present review addresses a complementary question: how securely can structurally characterized metabolites from these organisms be connected to the genes and enzymatic transformations responsible for their biosynthesis? We therefore focus primarily on BGC-associated metabolites reported after 2017, together with earlier natural products whose biosynthetic origins were resolved in subsequent studies. Representative examples are organized by genus and structural class, with particular attention to the experimental evidence supporting each BGC–metabolite relationship. Rather than providing an exhaustive inventory of marine rare-actinomycete metabolites, we distinguish biosynthetic assignments based principally on bioinformatic or metabolomic inference from those supported by genetic experiments, heterologous production, or biochemical characterization [11,15]. This biosynthesis-centered perspective highlights not only the chemical diversity of marine-derived rare actinomycetes, but also the pathway architectures and enzymatic transformations through which that diversity is generated.

## 2. BGC-Guided Discovery of Marine-Derived Rare Actinomycete Metabolites

BGC-guided studies begin with genomic prediction, but assigning a chemical product to a candidate locus remains an experimental problem. In studies of marine-derived rare actinomycetes, candidate clusters are typically nominated by sequence analysis and comparative genomics and then examined alongside LC–MS/MS metabolomics and molecular networking [16,17,18]. These approaches are particularly useful when pathway expression is low or absent under standard culture conditions [10,19]. Changes in cultivation, co-culture, targeted pathway activation, and heterologous expression can reveal products that are otherwise not detected [12,19]. Neither a cluster prediction nor a genome–metabolome correlation alone establishes biosynthetic origin.

Throughout this review, each BGC–metabolite assignment is considered according to the experiment that supports it. Sequence homology, domain organization, precursor-feeding data, and metabolomic correlation are treated as predictive or supportive evidence. Targeted gene inactivation and complementation establish genetic support. Heterologous production can directly connect a reconstructed locus with its product. Purified-enzyme assays test individual transformations and can define catalytic function or reaction order [15,19]. These distinctions are used in Section 3, Section 4, Section 5, Section 6, Section 7 and Section 8 to separate proposed biosynthetic associations from genetically supported pathways and biochemically established steps.

## 3. Marine-Derived Rare Actinomycetes: *Micromonospora*

### 3.1. Genus Overview and Biosynthetic Capacity

*Micromonospora* is one of the most extensively investigated genera of rare actinomycetes, and marine-derived strains have been recovered from diverse marine and marine-associated habitats, including deep-sea sediments, sponges, ascidians, and algal surfaces [20,21,22]. Genome sequencing and comparative genomic analyses have revealed that members of this genus harbor a diverse repertoire of biosynthetic gene clusters (BGCs), many of which remain unlinked to metabolites detectable under routine cultivation conditions [21,22,23]. Accordingly, recent efforts to discover natural products from marine-derived *Micromonospora* have increasingly relied on integrated BGC-guided strategies, including heterologous expression, targeted gene inactivation, co-culture-mediated pathway activation, metabolic flux redirection, and metabolomics-guided genome mining [24,25,26,27].

**Figure 1:**
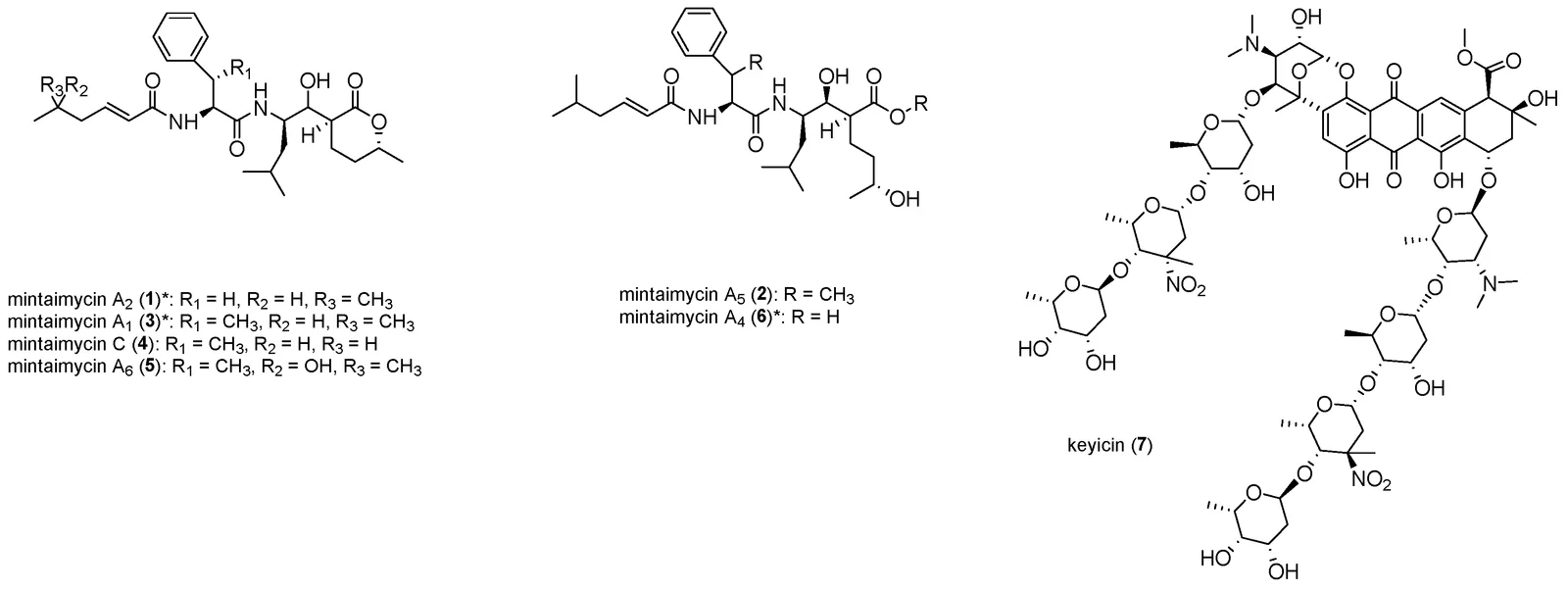

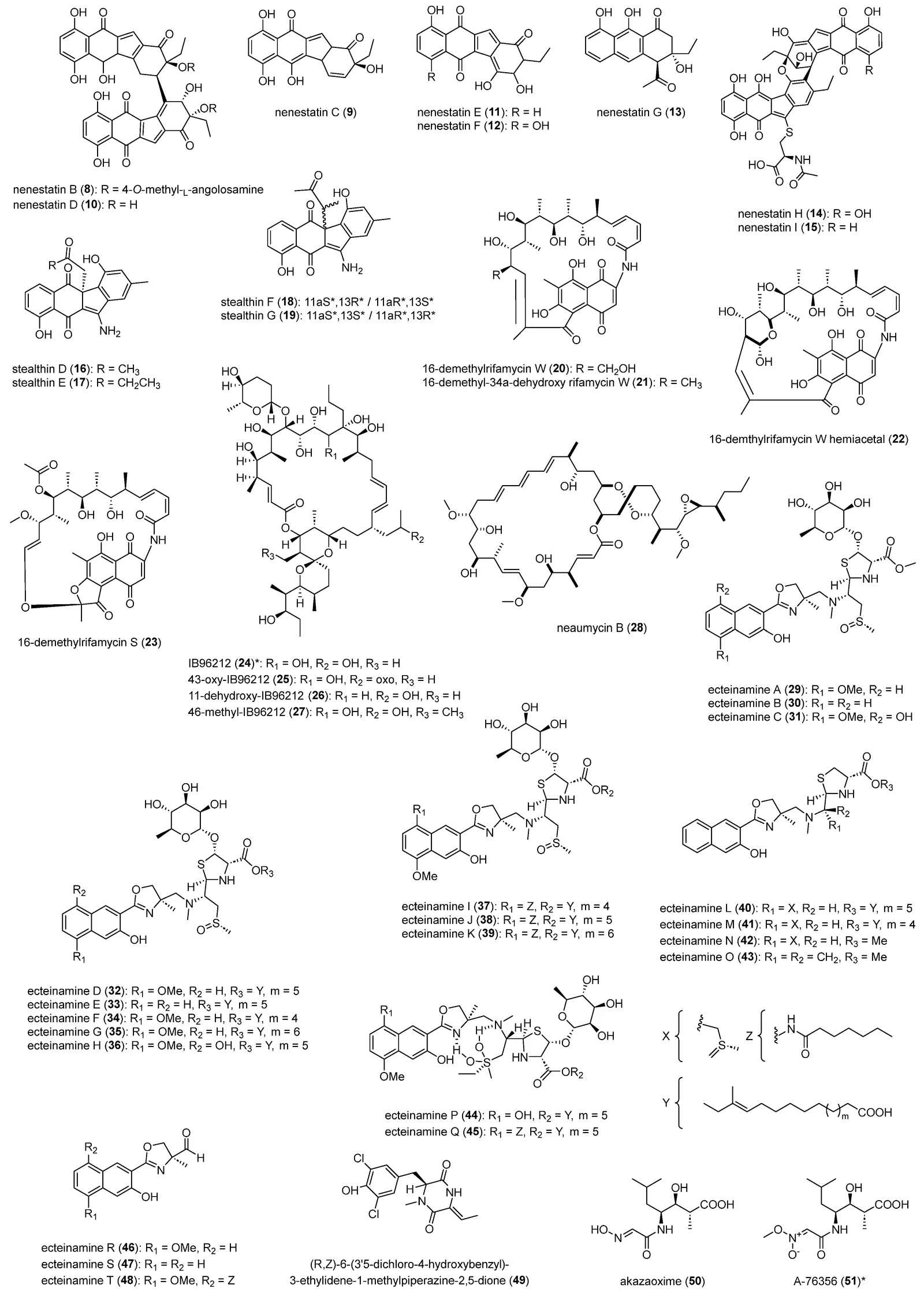
Structural overview of biosynthetic gene cluster (BGC)-linked metabolites from marine-derived *Micromonospora* species (*: previously reported compound).

The principal biosynthetic and structural classes discussed in the following subsections are polyketide synthase–nonribosomal peptide synthetase (PKS–NRPS) hybrid metabolites, type II PKS-derived anthracyclines and atypical angucyclines, type I PKS-derived ansamycins and spiroketal macrolides, and NRPS-derived peptidic metallophores [24,25,26,27,28,29,30,31,32,33,34]. A limited number of structurally characterized metabolites for which no cognate BGC has yet been assigned are also discussed. The corresponding chemical structures are presented in Figure 1, and the BGC-linked metabolites are summarized in Table 1.

### 3.2. PKS–NRPS Hybrid Metabolites: Mintaimycins

**Figure 2:**
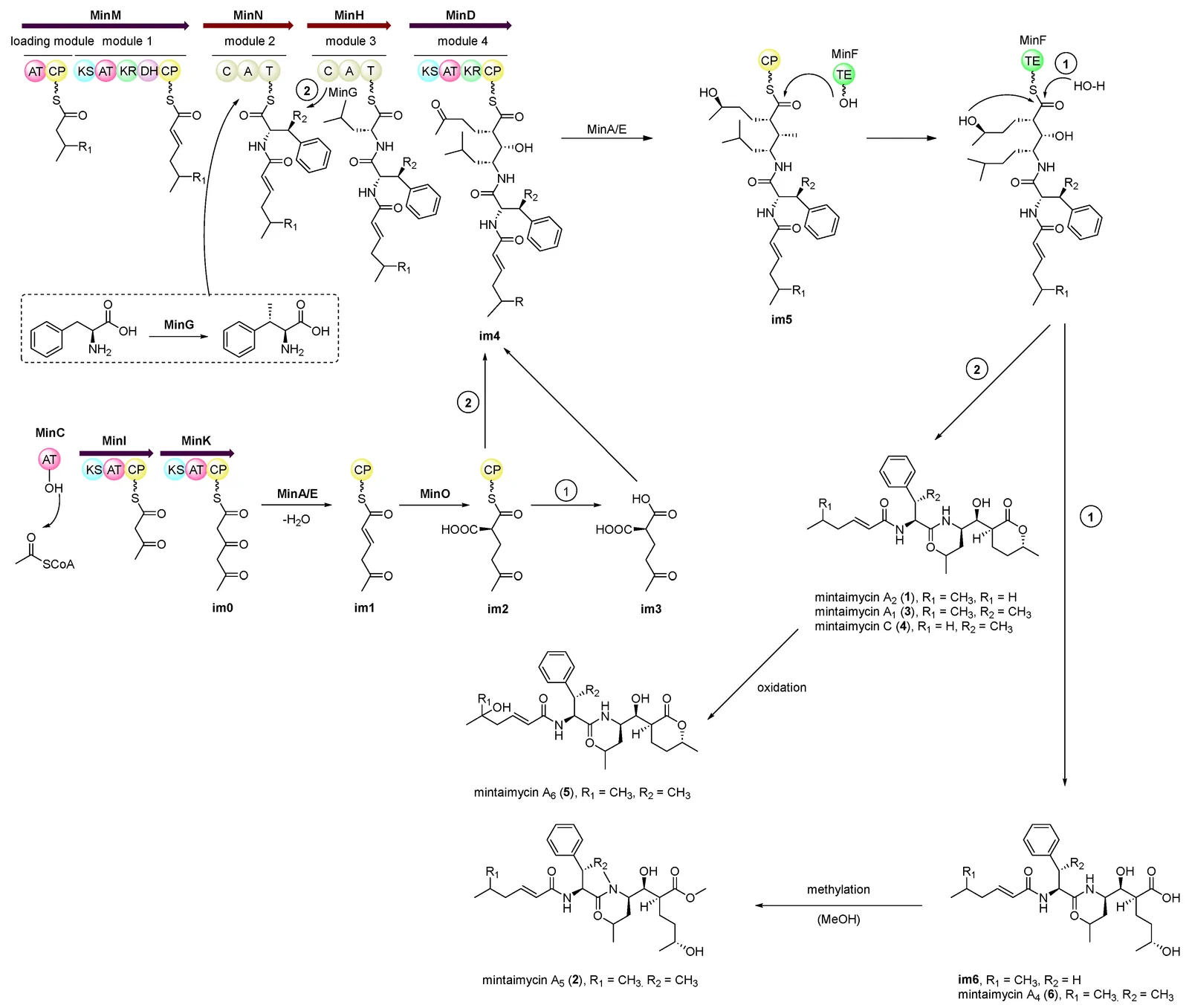
Proposed biosynthetic pathway of mintaimycins (**1**–**6**) in marine-derived *Micromonospora* sp. SCSIO 80128.

Mintaimycins serve as a robust example of BGC validation through heterologous expression. Mintaimycins A5 (**2**), C (**4**), and A6 (**5**) were reported as novel congeners from marine-derived *Micromonospora* sp. SCSIO 80128, together with the known mintaimycins A2 (**1**), A1 (**3**), and A4 (**6**). The *min* PKS–NRPS hybrid BGC was directly linked to mintaimycin biosynthesis by heterologous expression in *Streptomyces albus* J1074. Additional gene inactivation experiments supported the proposed functions of biosynthetic genes involved in β-methylation, chain release, and lactonization, and the stereochemistry of the mintaimycin family was revised accordingly [24]. The proposed biosynthetic pathway of mintaimycins (**1**–**6**) is presented in Figure 2. The mintaimycins did not exhibit detectable antibacterial activity against the tested strains. Among these metabolites, mintaimycin A1 (**3**) exhibited moderate anti-inflammatory activity at 20 µM and induced preadipocyte differentiation at 10 µM. However, it did not exhibit antitubercular or proangiogenic activity and was not cytotoxic to HeLa, HepG2, A549, or 4T1 cells [24].

### 3.3. Type II PKS Metabolites: Keyicin, Nenestatins, and Stealthins

Keyicin (**7**) exemplifies co-culture-mediated activation of a cryptic BGC. Although *Micromonospora* sp. WMMB-235 did not produce **7** under monoculture conditions, its biosynthesis was triggered upon co-culture with *Rhodococcus* sp. WMMA-185 [25]. Integrated transcriptomic and proteomic profiling in a subsequent multi-omics study indicated that a *Rhodococcus*-derived small-molecule signal activates the *Micromonospora*-encoded *kyc* type II PKS gene cluster. Thus, **7** provides an example of interspecies interaction-mediated cryptic BGC activation in marine-associated rare actinomycetes [25,28]. Keyicin (**7**) exhibited selective antibacterial activity against Gram-positive bacteria, with MIC values of 8 µg/mL (9.9 µM) against *Bacillus subtilis* and 2 µg/mL (2.5 µM) against methicillin-susceptible *Staphylococcus aureus* (MSSA). It also inhibited the growth of *Mycobacterium* sp. and *Rhodococcus* sp. [25].

Nenestatins illustrate how targeted gene inactivation can be used to interrogate dimerization and pathway branching in an atypical angucycline pathway. Inactivation of *nes18* in *Micromonospora echinospora* SCSIO 04089 abolished the production of dimeric nenestatin B (**8**) and led to the accumulation of monomeric nenestatin C (**9**), suggesting that the NmrA family protein Nes18 is involved in C–C dimerization [29]. The structure of 8 was established with the aid of nenestatin D (**10**), a deglycosylated aglycone generated by acid hydrolysis of **8**, which resolved the overlapping NMR signals of the parent compound. Although **10** was obtained semisynthetically rather than isolated as a natural product, it was proposed as the direct precursor of **8**, with two 4-*O*-methyl-l-angolosamine units attached at C-3 and C-3′ during the final glycosylation step [29]. A subsequent study using the Δ*nes5* mutant revealed that inactivation of the α/β hydrolase-like gene *nes5* abolished normal nenestatin A/B production and redirected the pathway toward nenestatins E–I (**11**–**15**). Together, these findings indicate how perturbation of the *nes* BGC can unravel tailoring and dimerization mechanisms in nenestatin biosynthesis [30]. Nenestatins B, C, and E–I (**8**, **9**, and **11**–**15**) did not exhibit antibacterial or cytotoxic activity under the reported assay conditions. No inhibition was observed against six bacterial strains at 64 µg/mL, and no cytotoxicity was detected against the SF-268, HepG-2, MCF-7, and A549 cell lines at concentrations up to 100 µM [29,30].

The fluostatin-related stealthins D–G (**16**–**19**) provide another example of gene inactivation-guided metabolite discovery. In marine-derived *Micromonospora rosaria* SCSIO N160, the inactivation of *flsN3*, which encodes a putative amidotransferase homologous to N–N bond-forming enzymes, altered the fluostatin metabolite profile and led to the accumulation of four novel racemic aminobenzo[*b*]fluorenes, stealthins D–G (**16**–**19**). As N–N bond-containing fluostatin metabolites were not detected in the Δ*flsN3* mutant, FlsN3 was proposed to participate in the formation or modification of nitrogen-containing fluostatins. However, its precise biochemical function remains unknown [31]. The biological activity of **16**–**19** was not evaluated. Nevertheless, their accumulation in the Δ*flsN3* mutant provides insight into the biosynthesis of N–N bond-containing fluostatins [31].

### 3.4. Type I PKS Metabolites: 16-Demethylrifamycins, IB-96212-Type Macrolides, and Neaumycin B

The 16-demethylrifamycins (**20**–**23**) exemplify metabolic flux redirection and biosynthetic pathway crosstalk. In *Micromonospora* sp. TP-A0468, deletion of the predominant *kst* polyketide pathway abolished kosinostatin production and enhanced the accumulation of the previously low-abundance 16-demethylrifamycins, namely 16-demethylrifamycin W (**20**), 16-demethyl-34a-dehydroxy rifamycin W (**21**), 16-demethylrifamycin W hemiacetal (**22**), and 16-demethylrifamycin S (**23**). Gene disruption, 3-amino-5-hydroxybenzoic acid (AHBA) complementation, and genome mining indicated that their biosynthesis requires functional crosstalk between two distantly located clusters, *pks3* and *M-rif*. The *pks3* cluster contributes to AHBA starter unit-related functions, whereas the *M-rif* cluster contributes to rifamycin backbone assembly [27]. Among these metabolites, **23** retained the antibacterial activity of rifamycin S, as it bears all structural moieties essential for activity. It exhibited MIC values of <0.25 µM against *S. aureus*, 1 µM against *B. subtilis*, 2 µM against *Acinetobacter baumannii*, 8 µM against *Mycobacterium smegmatis*, and 32 µM against *Escherichia coli*. In contrast, **22** exhibited only weak activity against *S. aureus* and *B. subtilis*, with MIC values of 128 and 256 µM, respectively, whereas **20** and **21** were inactive against all tested strains at concentrations up to 256 µM [27].

Marine-derived *Micromonospora* sp. FIMYZ51 produced three novel spiroketal macrolides, 43-oxy-IB-96212 (**25**), 11-dehydroxy-IB-96212 (**26**), and 46-methyl-IB-96212 (**27**), together with the known parent macrolide IB-96212 (**24**). These macrolides share an l-rhodinose moiety and a 6,6-spiroketal ring system. Genome mining and structural analysis supported a plausible PKS/spirocyclase-mediated biosynthetic pathway in which a vicenistatin-like type I PKS and an OlmO-like spirocyclase are proposed to assemble the macrocyclic core and generate the spiroketal moiety, respectively. However, as this pathway was not experimentally validated by gene inactivation or heterologous expression, the BGC assignment of **24**–**27** should be regarded as proposed and supported primarily by genome mining and structural analysis [32]. These macrolides exhibited potent antifungal activity against *Aspergillus niger*, with MIC values of 0.5–2.0 µg/mL, comparable to that of itraconazole (MIC 1 µg/mL). They also exhibited activity against *Candida albicans* (MIC 2–4 µg/mL) and the endocarditis-associated pathogen *Micrococcus luteus* (MIC 0.0625–1 µg/mL), with **26** exhibiting the strongest activity against *M. luteus*. All four macrolides also exhibited cytotoxicity against the drug-resistant tumor cell lines Namalwa and U266, with IC_50_ values ranging from 5.34 to 19.95 µM. Among them, **25** and **26** exhibited the greatest potency against Namalwa and U266 cells, with IC_50_ values of 5.34 and 8.91 µM, respectively [32].

For neaumycin B (**28**) from marine-derived *Micromonospora* sp. CNY-010, a *neu* type I PKS gene cluster was identified as the most plausible biosynthetic origin. Its full stereostructure, comprising 19 asymmetric centers, was elucidated by combining genomic information with NMR analysis, representing a genome-guided stereostructural elucidation approach rather than a fully validated biosynthetic pathway [33]. In the NCI-60 cell line panel, **28** exhibited pronounced selectivity toward the multiple myeloma cell line RPMI-8226. Replicate analyses also revealed potent activity against U87 human glioblastoma cells, with an LD_50_ value of 5.6 × 10^−5^ µg/mL (6.4 × 10^−8^ µM) [33].

### 3.5. NRPS Metabolites: Ecteinamines

**Figure 3:**
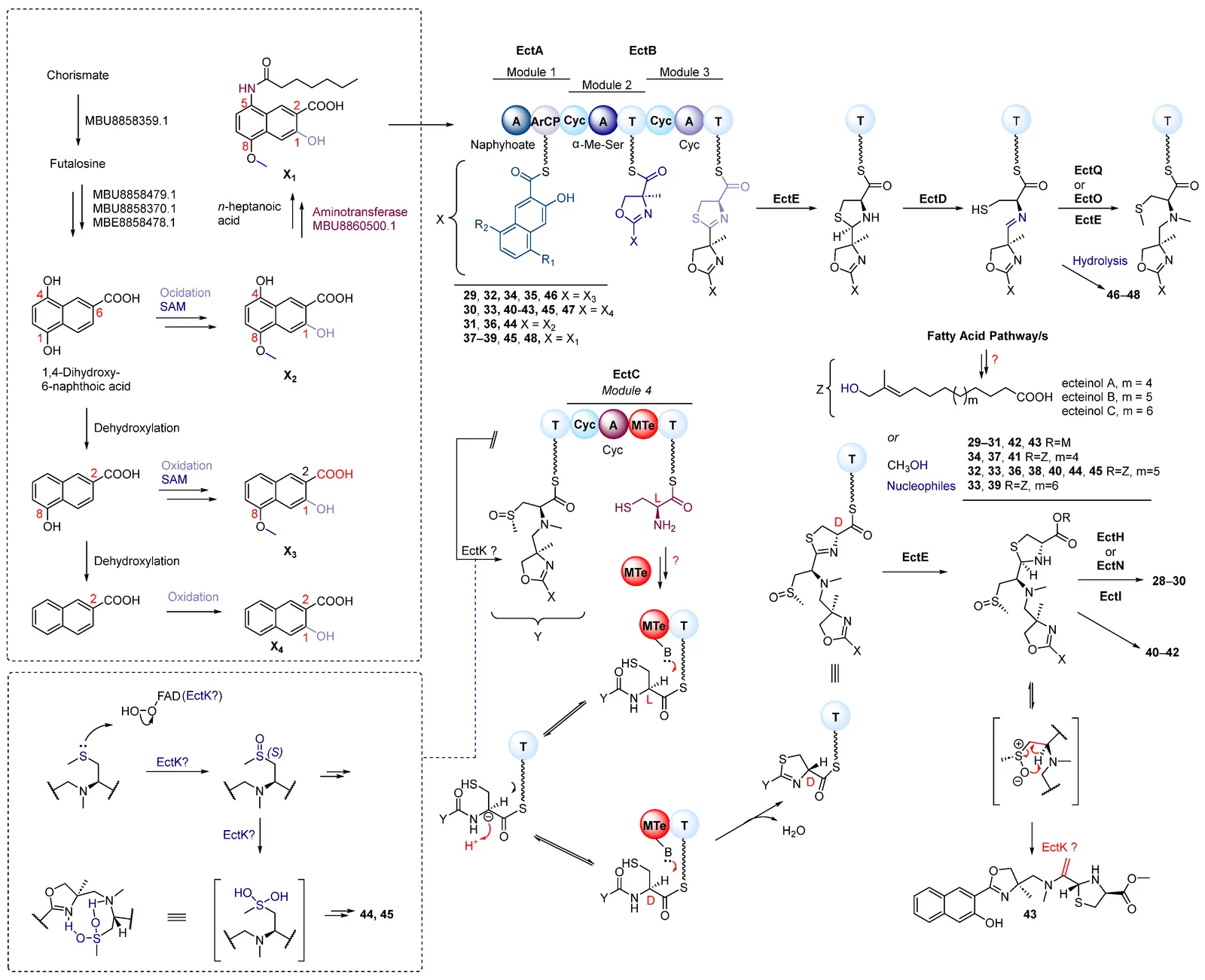
Proposed biosynthetic pathway of ecteinamines A–T (**29**–**48**) in marine-derived *Micromonospora* sp. WMMB482.

Ecteinamines A–T (**29**–**48**) exemplify an integrated metabolomic and genome mining approach for natural product discovery. The combined use of LC–MS-based hierarchical cluster analysis and principal component analysis (HCA–PCA) prioritization, GNPS molecular networking, genome mining, and isotope-feeding experiments led to the discovery of 20 novel ecteinamine-related compounds from *Micromonospora* sp. WMMB482. Ecteinamines A–Q (**29**–**45**) were characterized as intact nonribosomal peptidic metallophores, whereas ecteinamines R–T (**46**–**48**) were characterized as shunt products. Biosynthetic analyses suggested that the *ect* BGC was involved in the construction of the core scaffold, while a menaquinone-derived *mqn* pathway and unclustered tailoring genes contributed to the 2-naphthoate unit and late-stage modifications. As this pathway was not directly validated by gene inactivation or heterologous expression, it should be described as a proposed biosynthetic pathway [26]. The proposed biosynthetic pathway of ecteinamines A–T (**29**–**48**) is presented in Figure 3. Chrome azurol S and iron-regulation assays indicated that ecteinamine production was not induced by iron limitation, suggesting that these metabolites are not genuine siderophores. Instead, ecteinamine A (**29**) formed stable complexes with Ni, Co, Zn, and Cu, supporting its broad-spectrum metallophore function. No antibacterial or cytotoxic activity data were reported for ecteinamines A–T (**29**–**48**) [26].

### 3.6. Other Class Metabolites: Dichloro-Diketopiperazine, Akazaoxime, and A-76356

In addition to the polyketide- and nonribosomal peptide-derived metabolites described above, marine-derived *Micromonospora* species produce metabolites whose biosynthetic origins remain unassigned. A novel dichloro-diketopiperazine (**49**) was co-isolated with the IB-96212-type spiroketal macrolides from *Micromonospora* sp. FIMYZ51. A cognate BGC was not assigned to this halogenated cyclic dipeptide, which exhibited only weak antibacterial activity against *M. luteus* (MIC 32 µg/mL) [32].

In another study, marine-derived *Micromonospora* sp. AKA109 produced the enteromycin-class antibiotic akazaoxime (**50**), which bears an aldoxime functionality in place of the *O*-methyl nitronic acid, together with the known congener A-76356 (**51**). Because only a limited amount of **50** was available from the producing strain, its structure was elucidated by spectroscopic analyses of the natural product and corroborated by total synthesis, which supplied sufficient material for biological evaluation. Precursor-feeding experiments indicated that the carbon skeletons of **50** and **51** are derived from propionate (methylmalonate), leucine, and glycine. No cognate BGC was experimentally assigned. Therefore, **50** and **51** are regarded here as examples of structural diversity rather than as validated BGC-linked metabolites [34]. Akazaoxime (**50**) exhibited weak-to-moderate antimicrobial activity against the Gram-positive bacterium *Kocuria rhizophila* (MIC 50 µg/mL), whereas A-76356 (**51**) was only weakly active against the plant pathogen *Glomerella cingulata* (MIC 100 µg/mL). A synthetic intermediate and an unnatural analog prepared during the total synthesis exhibited MIC values of 50 µg/mL against *G. cingulata* and 25–50 µg/mL against the human pathogen *Trichophyton rubrum* [34].

## 4. Marine-Derived Rare Actinomycetes: *Nocardiopsis*

### 4.1. Genus Overview and Biosynthetic Capacity

*Nocardiopsis* is a halotolerant actinomycete genus frequently recovered from saline and marine-associated environments, including marine sediments and sponge-derived microbial communities [35,36,37,38]. Genome analyses have revealed a broad complement of BGCs across the genus, and marine-derived strains have been reported to produce structurally diverse secondary metabolites, including aromatic polyketides, macrolactams, and diketopiperazine (DKP) alkaloids [10,37,38,39,40]. BGC-linked discovery in this genus has relied on chemical elicitation of cryptic antibacterial activity, draft genome sequencing combined with targeted gene deletion, and enzyme-level reconstitution of tailoring steps [38,41,42,43].

**Figure 4:**
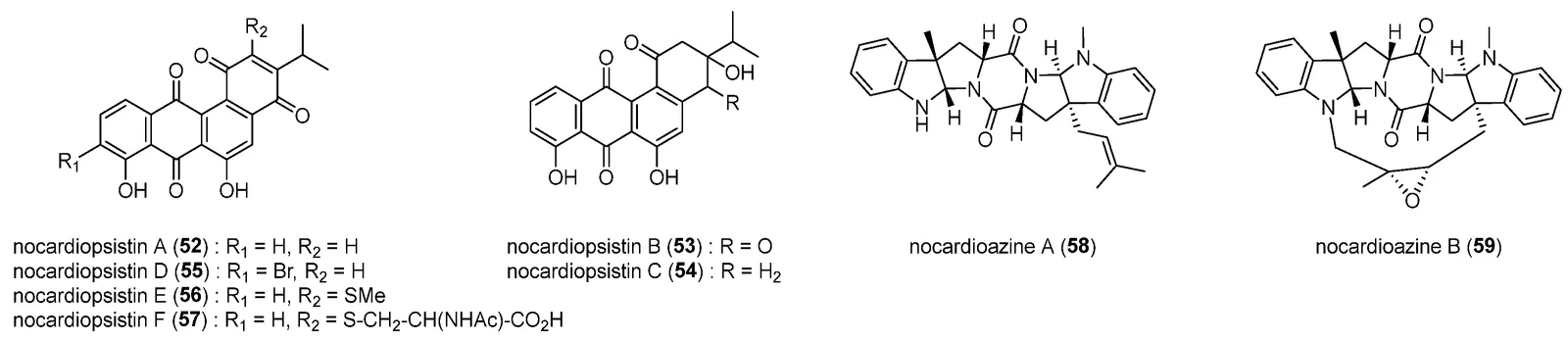
Structural overview of biosynthetic gene cluster (BGC)-linked metabolites from marine-derived *Nocardiopsis* species.

The principal biosynthetic and structural classes discussed in the following subsections are type II PKS-derived angucycline aromatic polyketides [38,41] and cyclodipeptide synthase (CDPS)-derived prenylated DKP alkaloids [40,42,43]. The corresponding chemical structures are presented in Figure 4, and the BGC-linked metabolites are summarized in Table 2.

### 4.2. PKS Metabolites: Nocardiopsistins

**Figure 5:**
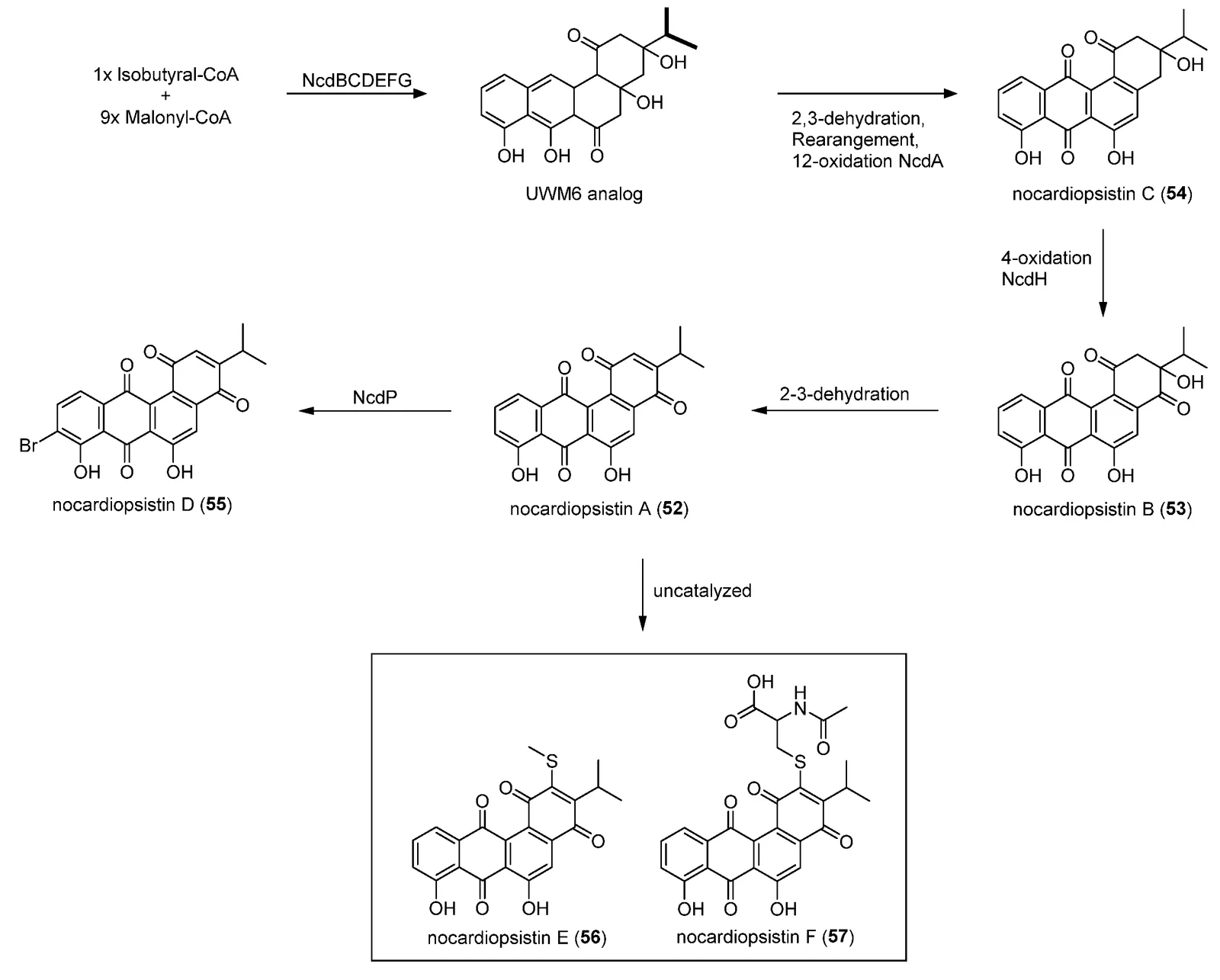
Proposed biosynthetic pathway of nocardiopsistins A–F (**52**–**57**) in marine sponge-derived *Nocardiopsis* sp. HB-J378.

Nocardiopsistins provide a clear example in which novel metabolites were directly linked to a BGC. Draft genome sequencing and antiSMASH analysis of sponge-derived *Nocardiopsis* sp. HB-J378 predicted 23 BGCs, among which a type II PKS gene cluster (*ncd*) containing the core genes encoding KSα, KSβ/CLF, and ACP was identified as the candidate locus [38]. In-frame deletion of the PKS cassette *ncdCDE* abolished the production of nocardiopsistins A–C (**52**–**54**) and eliminated the anti-MRSA activity of the mutants, confirming that this cluster is essential for their biosynthesis. The pathway was proposed to proceed through an analog of the angucycline intermediate UWM6, assembled from an unusual isobutyryl-CoA starter unit and nine malonyl-CoA extender units. The use of isobutyryl-CoA, which is rarely employed as a starter unit in angucycline biosynthesis, distinguishes this pathway from canonical angucycline routes [38]. The proposed biosynthetic pathway of nocardiopsistins A–F (**52**–**57**) is presented in Figure 5. Nocardiopsistins A–C (**52**–**54**) inhibited MRSA, with nocardiopsistin B (**53**) being the most potent, exhibiting an MIC value of 3.12 µg/mL, equal to that of chloramphenicol. Nocardiopsistins A and C (**52** and **54**) were moderately active, with MIC values of 12.5 µg/mL, whereas none of the three metabolites inhibited *C. albicans* or *Pseudomonas aeruginosa*. Supplementation with 2 mM LaCl_3_ as a chemical elicitor doubled the anti-MRSA activity of the producing strain, illustrating how elicitation can activate or enhance cryptic metabolite production in marine-derived rare actinomycetes [38].

Three additional congeners were subsequently characterized from the same strain and proposed to arise from the same *ncd* pathway, with nocardiopsistin A (**52**) serving as the common precursor. These metabolites comprised nocardiopsistin D (**55**), the first brominated angucycline natural product, together with the sulfur-containing nocardiopsistins E and F (**56** and **57**) [41]. NcdP, a putative flavin-dependent halogenase, was implicated in the C-9 bromination of **52** to yield **55**. Overexpression of *ncdP* increased the ratio of **55** to **52**, supporting its proposed role in bromination. Its enzymatic activity has not been directly validated. No genes potentially involved in C–S bond formation were identified within the *ncd* cluster or the surrounding genomic region. The methylthio substituent of nocardiopsistin E (**56**) was therefore proposed to arise through the nonenzymatic conjugate addition of methanethiol. Nocardiopsistin F (**57**) was proposed to form through the nonenzymatic addition of *N*-acetylcysteine or through incorporation involving mycothiol [41].

The introduction of a single bromine atom markedly enhanced and broadened the antibacterial activity. Nocardiopsistin D (**55**) inhibited MRSA at 0.098 µg/mL, representing a 128-fold improvement over nocardiopsistin A (**52**). It was also active against two vancomycin-resistant *S. aureus* strains, VRSA 170 and 522, with MIC values of 0.031 µg/mL, as well as against *Enterococcus faecium* and *B. cereus*, with MIC values of 0.031 and 0.063 µg/mL, respectively. In comparison, **52** exhibited substantially weaker activity against these strains, with MIC values of 6.25–12.5, >50, and 12.5 µg/mL, respectively. Nocardiopsistins E and F (**56** and **57**) inhibited MRSA at 3.125 and 0.195 µg/mL, respectively. Bromination did not substantially alter cytotoxicity, as **55** and **52** exhibited comparable IC_50_ values against Vero cells, 7.1 and 10 µM, respectively, and HepG2 cells, 9.3 and 2.6 µM, respectively. These findings indicate that **55** is considerably more potent as an antibacterial agent than as a cytotoxic compound [41].

### 4.3. Other Class Metabolites: Nocardioazines

Nocardioazines represent a complementary case in which the natural products were discovered before their biosynthetic basis was elucidated. Subsequent studies established the complete biosynthetic pathway to nocardioazine B (**59**), whereas the conversion of **59** to nocardioazine A (**58**) remains unresolved. **58** and **59** were initially reported from the Australian marine sediment-derived strain *Nocardiopsis* sp. CMB-M0232. The saline cultures of this strain were rich in DKPs, and **58** and **59** were isolated together with cyclo(l-Trp–l-Trp) and cyclo(l-Trp–d-Trp). They were proposed as the first *C*-prenylated DKPs reported from a marine-derived bacterium and the first examples of normal C-3-prenylated DKPs from any source, with cyclo(l-Trp–l-Trp) suggested as a key biosynthetic precursor [40]. Meanwhile, **58** and **59** did not exhibit antibacterial activity against the tested Gram-positive and Gram-negative bacteria or cytotoxicity against the tested human cancer cell lines. Nocardioazine A (**58**) was nevertheless identified as a noncytotoxic inhibitor of the efflux pump P-glycoprotein (P-gp). At 20 µM, **58** increased intracellular calcein fluorescence in P-gp-overexpressing SW620 Ad300 colon cancer cells and reversed doxorubicin resistance. Nocardioazine B (**59**) did not exhibit P-gp inhibitory activity. The absence of inhibition by **59** suggests that the bridged DKP scaffold of **58** is important for this activity [40].

A subsequent study identified two CDPS genes, *nozA* and *ncdA*, in the genome of *Nocardiopsis* sp. CMB-M0232. Although NozA and NcdA are phylogenetically distinct enzymes, heterologous expression in *E. coli* showed that both enzymes produce cyclo(l-Trp–l-Trp). The identity and stereochemical configuration of the product were confirmed by comparison with synthetic standards, tandem mass spectrometry (MS/MS), high-resolution mass spectrometry, and Marfey’s analysis. *In vitro* assays with purified recombinant enzymes further revealed that NozA and NcdA specifically use tryptophanyl-tRNA rather than free tryptophan or other aromatic aminoacyl-tRNAs [42].

More recently, the complete tailoring pathway leading to nocardioazine B (**59**) was elucidated. This study revealed that its biosynthesis is distributed across multiple genomic loci rather than encoded by a single contiguous BGC. The *noz* and *ncd* loci each encode a CDPS capable of producing the cyclo(l-Trp–l-Trp) precursor, whereas a separate *noz2* locus encodes the tailoring enzymes NozR, NozPT, and NozMT. Chemical complementation of *Streptomyces lividans* transformants carrying *noz2* with cyclo(l-Trp–l-Trp) resulted in **59** production. Co-expression of *noz* and *noz2* also yielded **59**. Neither *noz* nor *noz2* alone produced **59** *de novo* [43].

The first tailoring step is catalyzed by NozR, an unusual DKP d/L isomerase related to the Asp/Glu racemase family. This stereoisomerization is required because NozA and NcdA generate cyclo(l-Trp–l-Trp), whereas **58** and **59** possess a d,d-DKP configuration. NozR converts cyclo(l-Trp–l-Trp) into cyclo(d-Trp–l-Trp) and cyclo(d-Trp–d-Trp). Substitution of the proposed catalytic residues Cys-75 and Cys-179 abolished its stereoisomerase activity. NozPT, a phytoene synthase-like prenyltransferase, subsequently catalyzes C-3′ prenylation of cyclo(d-Trp–d-Trp) using dimethylallyl pyrophosphate (DMAPP) as the prenyl donor. Unlike related DKP prenyltransferases that generally act on l-configured substrates, NozPT accepts a d-configured DKP substrate. Finally, the *S*-adenosylmethionine (SAM)-dependent methyltransferase NozMT completes the biosynthesis of **59** through sequential N-1′ methylation, C-3 methylation, and annulation. *In vitro* assays and time-course experiments detected the N-1′-methylated intermediate before the formation of **59**, supporting the proposed reaction sequence and establishing NozMT as a rare dual-function methyltransferase that methylates two distinct nucleophilic sites within the same substrate [43]. The enzyme and genomic locus responsible for the subsequent conversion of nocardioazine B (**59**) into the bridged nocardioazine A (**58**) remain unidentified.

## 5. Marine-Derived Rare Actinomycetes: *Verrucosispora*

### 5.1. Genus Overview and Biosynthetic Capacity

*Verrucosispora* is a marine-associated rare actinomycete genus that has attracted attention for its capacity to produce structurally unusual polyketides and siderophores [44,45,46,47,48,49,50]. Genome analyses of marine-derived strains have revealed hybrid type I/type III PKS gene clusters that elaborate a 3,5-dihydroxybenzoic acid (3,5-DHBA) starter unit into carbacyclic ansa scaffolds. This biosynthetic logic remains comparatively underexplored among actinomycetes [44,46,48]. BGC-linked discovery in this genus has combined gene disruption, chemical complementation, heterologous expression, and *in vitro* enzymatic characterization with genome mining of cryptic, bioassay-silent metabolites [46,47,48,49,50].

**Figure 6:**
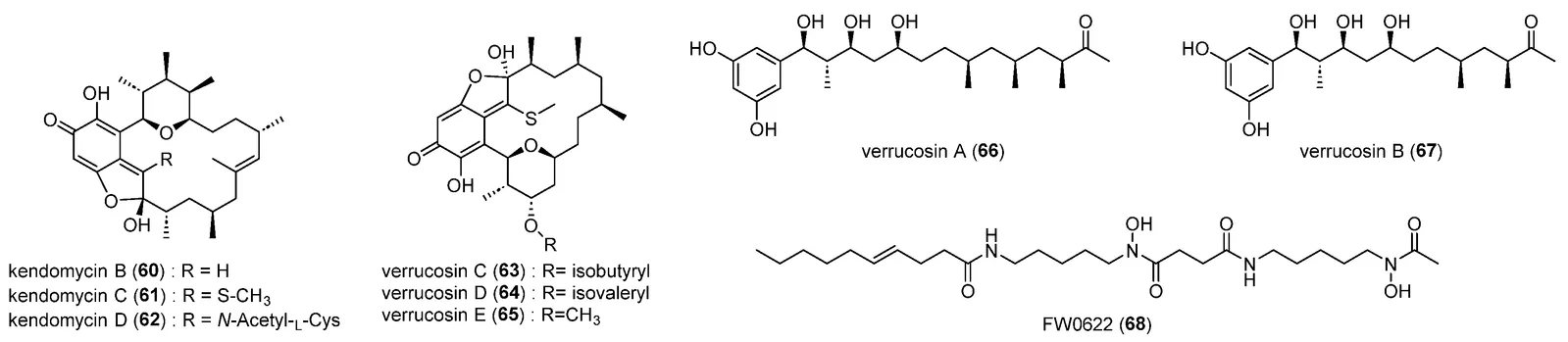
Structural overview of biosynthetic gene cluster (BGC)-linked metabolites from marine-derived *Verrucosispora* species.

The principal biosynthetic and structural classes discussed in the following subsections are hybrid type I/type III PKS-derived carbacyclic ansa and linear polyketides, together with a hydroxamate siderophore assembled by an NRPS-independent siderophore (NIS) synthetase pathway. The corresponding chemical structures are presented in Figure 6, and the BGC-linked metabolites are summarized in Table 3.

### 5.2. Type I/III Hybrid PKS Metabolites: Kendomycins and Verrucosins

Kendomycins provide the most extensively validated biosynthetic example from this genus. Kendomycins B–D (**60**–**62**), novel ansamycin-like polyketides, were isolated from the deep-sea sediment-derived strain *Verrucosispora* sp. SCSIO 07399 [44]. Unlike classical macrolactam-type ansamycins, these metabolites possess a carbacyclic ansa scaffold in which a highly substituted tetrahydropyran ring is linked to a quinone-type aromatic core. Kendomycin B (**60**) was proposed as the bona fide natural product of this strain, whereas kendomycins C and D (**61** and **62**) were proposed to be C-20 thiol-substituted adducts bearing a thiomethyl group and an *N*-acetyl-l-cysteine moiety, respectively [44]. Kendomycins B–D (**60**–**62**) inhibited six Gram-positive bacterial strains, including MRSA shhs-A1, with MIC values of 0.5–8.0 µg/mL. Moreover, **60** and **61** exhibited activity comparable to or greater than that of vancomycin, whereas **62** was one- to fourfold less active. These metabolites also exhibited cytotoxicity against six human tumor cell lines, MGC803, A549, HeLa, HepG2, MCF-7, and RKO, with IC_50_ values ranging from 2.2 to 44 µM. Comparable activity against the nonmalignant L02 and HUVEC-12 cell lines indicated limited selectivity toward tumor cells [44]. Kendomycin-type polyketides are not restricted to *Verrucosispora*. The parent kendomycin and kendomycin E were also isolated from *Streptomyces* strains. Kendomycin E, obtained from *Streptomyces* sp. Cl 58-27, bears distinct ansa-chain modifications and did not exhibit antimicrobial activity against the tested bacterial, yeast, or fungal strains [45].

Genome sequencing and antiSMASH analysis of *Verrucosispora* sp. SCSIO 07399 identified the *kmy* BGC, an approximately 80 kb hybrid type I/type III PKS cluster comprising 33 open reading frames and showing high similarity to the previously reported kendomycin cluster *ken* [48,49]. Within the *kmy* cluster, *kmy11* and *kmy18* were annotated as type I and type III PKS genes, respectively, and disruption of either gene abolished kendomycin B (**60**) production. Chemical complementation of the Δ*kmy18* mutant with 3,5-DHBA restored **60** production, supporting 3,5-DHBA as the starter unit. Heterologous expression of the complete *kmy* cluster in *Streptomyces coelicolor* M1152 resulted in the production of kendomycins B–D (**60**–**62**), confirming the involvement of this BGC in their biosynthesis [48].

**Figure 7:**
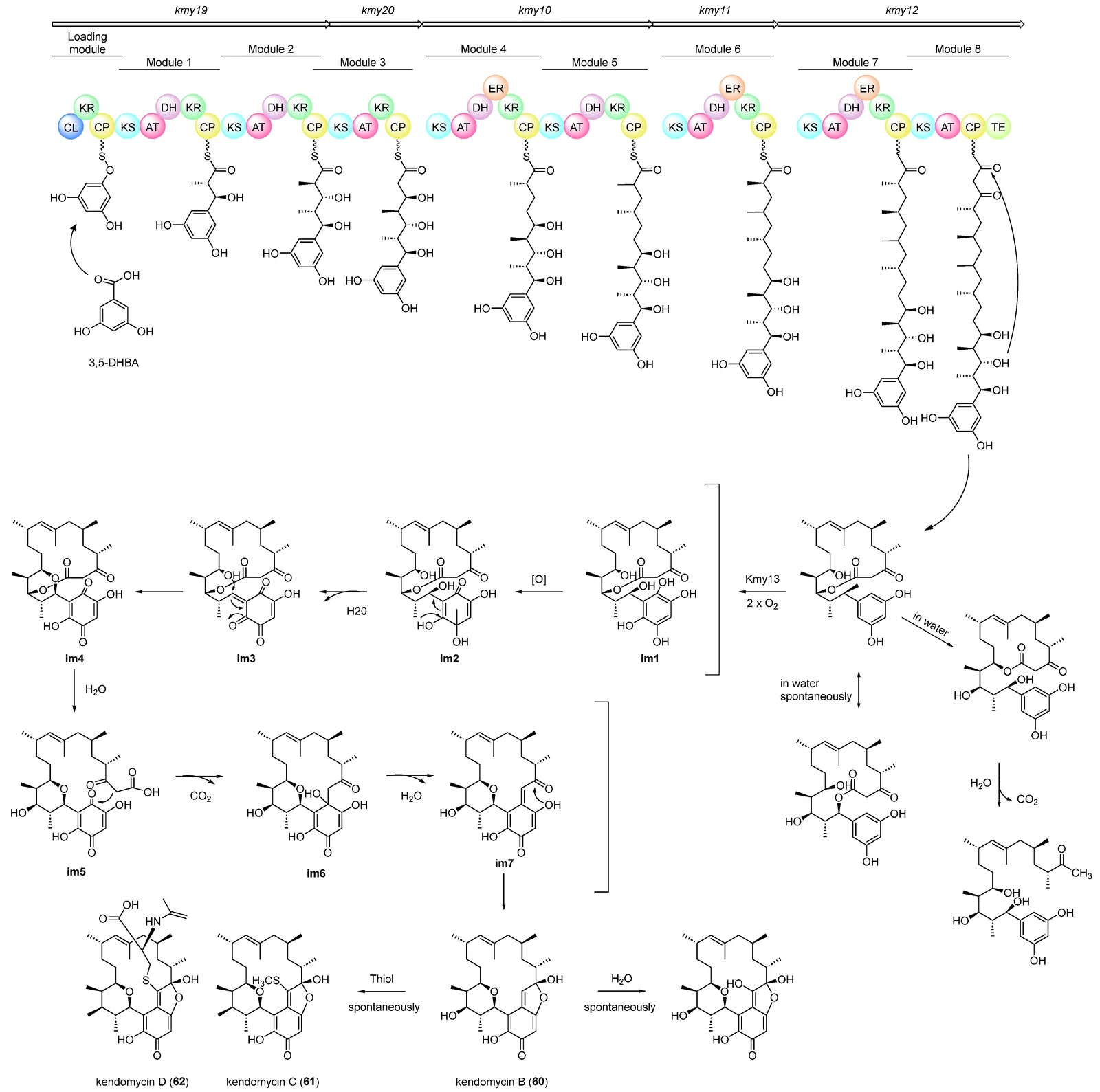
Proposed biosynthetic pathway of kendomycins B–D (**60**–**62**) in the deep-sea sediment-derived *Verrucosispora* sp. SCSIO 07399.

The kendomycin B (**60**) pathway is proposed to begin with type III PKS-mediated formation of the 3,5-DHBA starter unit, followed by chain elongation through a modular type I PKS assembly line. The resulting β-keto ester-containing intermediate is then converted into the characteristic carbacyclic ansa scaffold via post-PKS oxidative cyclization. In a recent mechanistic study, the FAD-dependent monooxygenase Kmy13 was identified as the key enzyme responsible for this transformation. Inactivation of *kmy13* resulted in the accumulation of kendolactones A–C. *In vitro* enzymatic assays revealed that Kmy13 converts kendolactone A into kendomycin B (**60**) in the presence of FAD and NADPH [50]. Thus, the *kmy* pathway represents a highly validated biosynthetic system supported by gene disruption, chemical complementation, heterologous expression, and enzymatic characterization. The proposed biosynthetic pathway of kendomycin B (**60**) is presented in Figure 7.

Verrucosins provide a proposed example in which a single BGC may generate two distinct scaffold types. Five novel polyketides were isolated from the marine-derived strain *Verrucosispora* sp. TAA-831. Verrucosins A and B (**66** and **67**) are linear polyketides structurally related to the baulamycins, whereas verrucosins C–E (**63**–**65**) are macrocyclic ansa polyketides related to kendomycin [46]. The conserved arrangement of malonate- and methylmalonate-derived units across the linear and macrocyclic congeners suggested a common biosynthetic origin. Targeted genome mining identified a hybrid type I/type III PKS BGC, *vrs*, as the most likely biosynthetic locus. This pathway was proposed to use a 3,5-DHBA-derived starter unit.

In the proposed pathway, a modular type I PKS assembly line generates a common polyketide intermediate. The FAD-dependent monooxygenase Vrs21 was proposed to facilitate oxidation and subsequent cyclization to form verrucosins C–E (**63**–**65**). Verrucosins A and B (**66** and **67**) were proposed to arise either through hydrolysis and subsequent decarboxylation of a putative macrolactone intermediate or through direct decarboxylative off-loading from the PKS assembly line. Verrucosin B (**67**), which lacks one methylmalonyl-derived extension unit relative to verrucosin A (**66**), was further proposed to result from module skipping [46]. Because the assignment of the *vrs* pathway is based on genome mining, BGC comparison, and structural relationships rather than genetic or biochemical validation, it should be regarded as proposed and distinguished from the experimentally validated *kmy* pathway.

These metabolites did not exhibit antibacterial activity against methicillin-resistant *S. aureus* TCH1516 or *E. coli* LptD4213 in disk diffusion assays at 10 µg per disk. Their significance therefore lies primarily in their structural novelty and in the proposed biosynthetic relationship between linear and macrocyclic polyketides [46].

### 5.3. Other Class Metabolites: FW0622

Beyond polyketides, marine-derived *Verrucosispora* species also produce siderophore-type metabolites. FW0622 (**68**), a novel desferrioxamine-like siderophore, was discovered from *Verrucosispora* sp. FIM060022 through draft genome-guided mining [47]. Comparative analysis of the putative siderophore BGC suggested that **68** is assembled through an NRPS-independent siderophore (NIS) synthetase pathway involving DesA–DesD-like enzymes. In the proposed pathway, lysine undergoes decarboxylation and hydroxylation to form *N*-hydroxycadaverine, followed by acylation and nucleoside triphosphate (NTP)-dependent condensation. FW0622 (**68**) did not exhibit antimicrobial activity against the tested microorganisms, including *S. aureus*, *B. subtilis*, *M. luteus*, *E. coli*, *C. albicans*, and *A. niger* (MIC > 10 mg/mL). Nevertheless, its discovery illustrates how genome mining can reveal cryptic metabolites that are not detected through conventional bioactivity-guided screening and demonstrates that the biosynthetic capacity of *Verrucosispora* extends beyond polyketide pathways [47].

## 6. Marine-Derived Rare Actinomycetes: *Salinispora*

### 6.1. Genus Overview and Biosynthetic Capacity

*Salinispora* is an obligately marine actinomycete genus widely distributed in tropical and subtropical marine sediments and has become a model system for biosynthetic gene cluster (BGC)-guided natural product discovery [7]. Comparative genomic analyses have identified a diverse repertoire of BGCs across the genus, many of which remain unlinked to characterized metabolites or are poorly expressed under standard cultivation conditions [51]. Various approaches, including comparative genomics, pattern-based genome mining, molecular networking, metabolomic analysis, and targeted gene inactivation, have been employed to establish or propose BGC–metabolite relationships in *Salinispora* [51,52,53,54,55,56].

**Figure 8:**
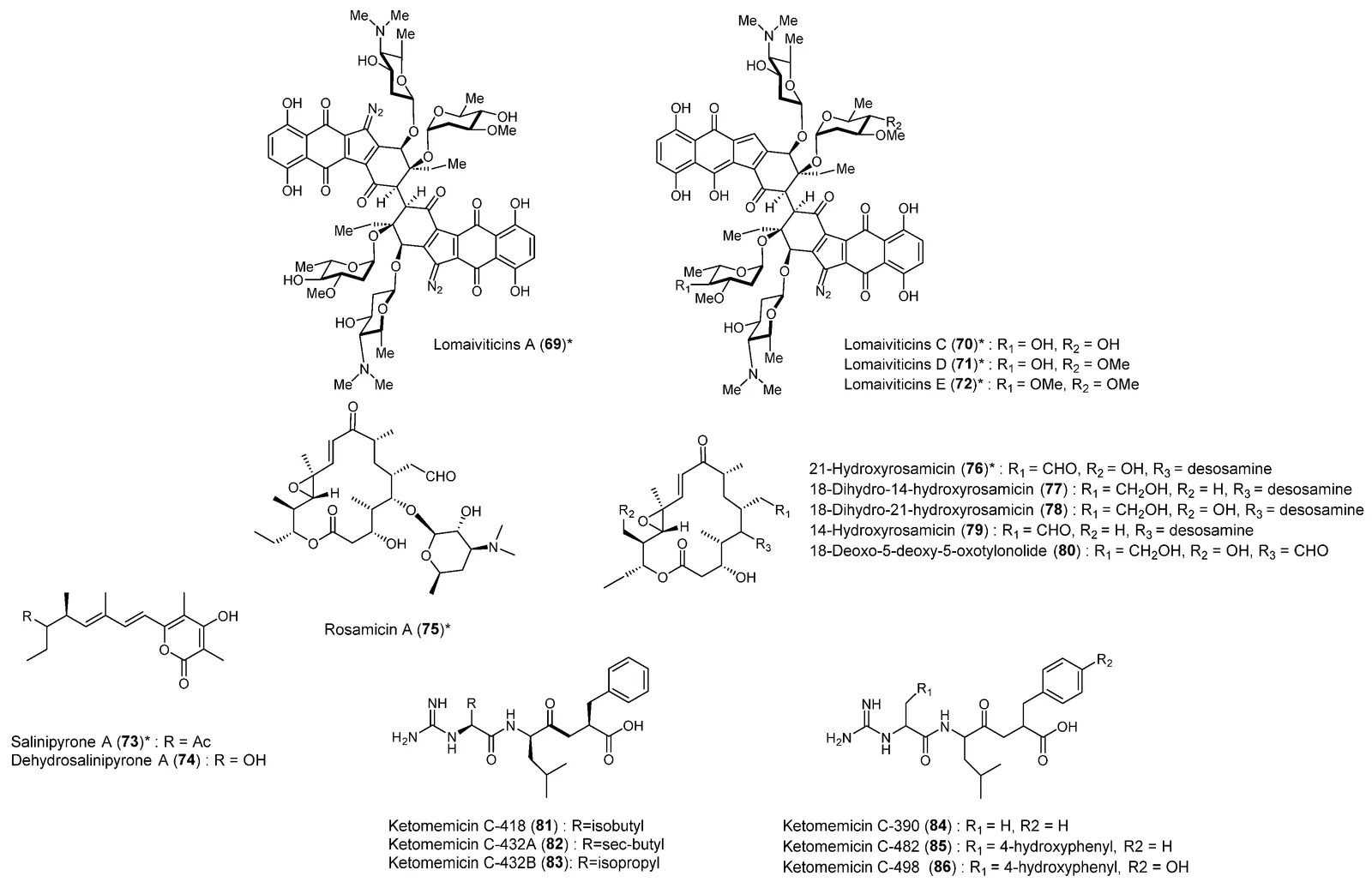
Structural overview of BGC-associated and putatively annotated metabolites from marine-derived *Salinispora* species (*: previously reported compound).

The metabolites discussed below include ATP-grasp peptide ligase-dependent, NRPS-independent pseudopeptides [52], type I PKS-derived macrolides and related pathway byproducts [55,56], and type II PKS-derived diazofluorene aromatic polyketides [53,54]. Their structures are shown in Figure 8, and the corresponding BGC assignments are summarized in Table 4.

### 6.2. Type II PKS Metabolites: Lomaiviticins A, C–E

Lomaiviticins A and C–E (**69**, **70**–**72**) are type II PKS-derived diazofluorene aromatic polyketides isolated from *Salinispora pacifica* strains DPJ-0016 and DPJ-0019. Lomaiviticin C (**70**) is C1-symmetric and contains a hydroxyfulvene unit in place of one diazofluorene moiety. Lomaiviticin D (**71**) was obtained as an inseparable mixture of two mono-*O*-methylated positional isomers, whereas **72** contains two additional *O*-methyl substituents on the oleandrose residues [57]. Moreover, **70**–**72** showed submicromolar antiproliferative activity against K562, LNCaP, HCT-116, and HeLa cells. Their IC_50_ values were 223–589 nM for **70**, 161–197 nM for **71**, and 255–964 nM for **72**. Lomaiviticin A (**69**) was substantially more potent, with IC_50_ values of 2–11 nM. This comparison suggests that the intact dimeric diazofluorene scaffold contributes substantially to antiproliferative potency. Synthetic conversion of **70** into **69** further established their direct structural relationship [57].

**Figure 9:**
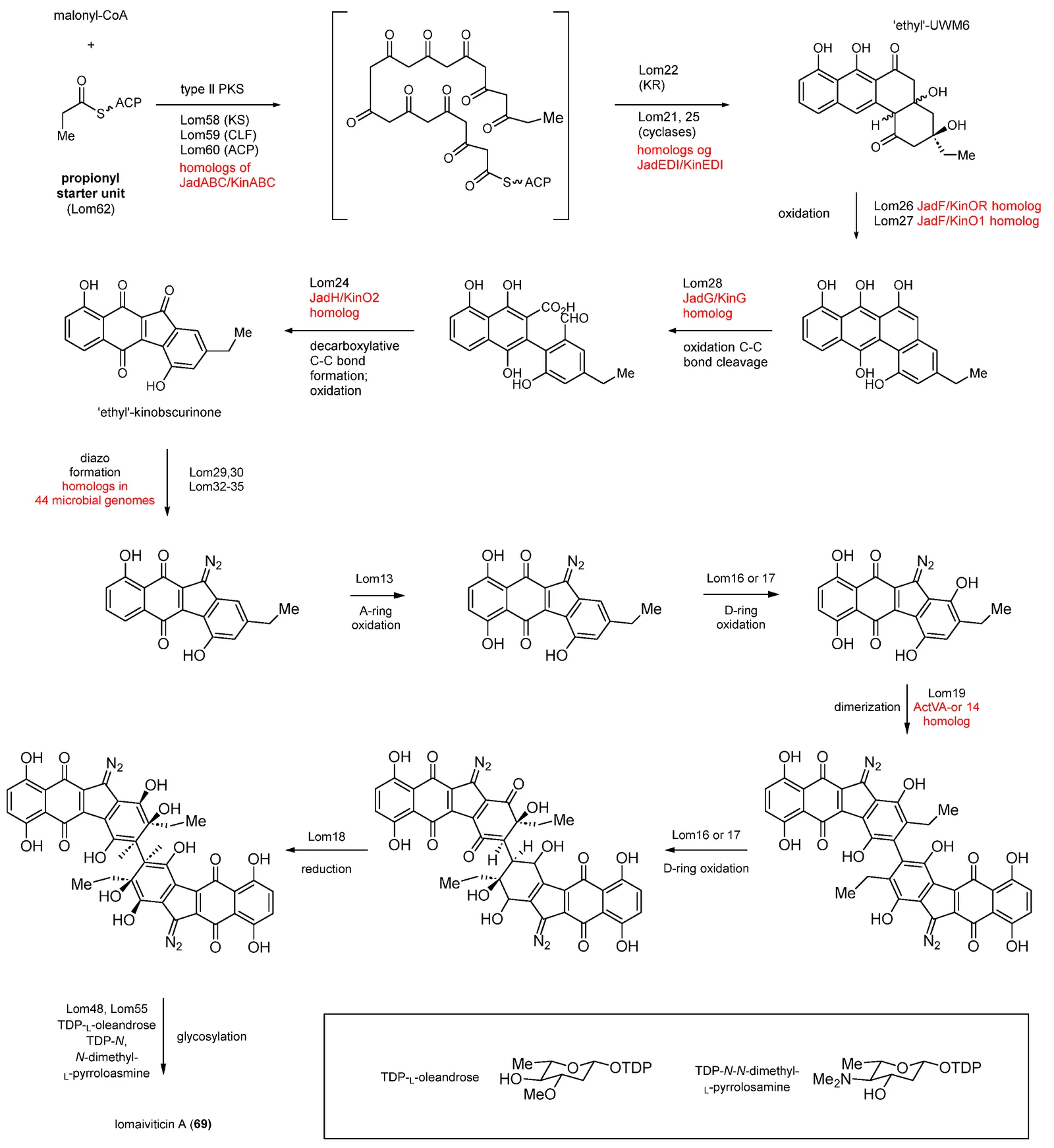
Proposed biosynthetic pathway of lomaiviticin A (**69**) in *Salinispora pacifica* DPJ-0016 and DPJ-0019.

Targeted disruption of the ketosynthase gene *lom58* abolished lomaiviticin production, genetically confirming the involvement of the *lom* type II PKS cluster. Cluster annotation identified candidate enzymes for diazo formation, dimerization, oxidation, and glycosylation, although the functions and reaction order of several late-stage tailoring enzymes remain unresolved [53]. The proposed pathway to **69** is shown in Figure 9.

### 6.3. Type I PKS Metabolites: Salinipyrone A, Dehydrosalinipyrone A, Rosamicin A and Its Derivatives

Salinipyrone A (**73**), dehydrosalinipyrone A (**74**), rosamicin A (**75**), and the rosamicin analog 21-hydroxyrosamicin (**76**), 18-dihydro-14-hydroxyrosamicin (**77**), 18-dihydro-21-hydroxyrosamicin (**78**), and 14-hydroxyrosamicin (**79**) were isolated from *Salinispora pacifica* CNS-237 [55]. Salinipyrone A (**73**) and dehydrosalinipyrone A (**74**) are pyrone polyketides, whereas rosamicin A (**75**) and its derivatives (**76**–**79**) are 16-membered macrolides. Both groups originate from the same modular type I PKS assembly line through differential module- and domain-skipping events [56].

Rosamicin derivatives (**76**–**79**) showed moderate antibacterial activity. Salinipyrone A (**73**) and dehydrosalinipyrone A (**74**) showed no appreciable antibacterial activity in the initial screen, although Salinipyrone A (**73**) inhibited interleukin-5 production by 50% at 10 μg/mL without measurable cytotoxicity toward HCT-116 cells [55].

Targeted inactivation of the *spr* PKS abolished production of both salinipyrones and rosamicins, genetically linking these metabolites to the *spr* type I PKS pathway. Mutation of the Spr10 ketoreductase domain further showed that rosamicins require the complete eight-module assembly line, whereas salinipyrones arise through module and domain skipping. The *spr10*-Y1290F mutant also accumulated 18-deoxo-5-deoxy-5-oxotylonolide (**80**), supporting the proposed pathway model [56].

### 6.4. Other Class Metabolites: Ketomemicin Derivatives

Ketomemicins C-418, C-432A, and C-432B (**81**–**83**) are ketomethylene-containing pseudopeptides isolated from *Salinispora pacifica* CNY-498. Related ketomemicins C-390, C-482, and C-498 (**84**–**86**) were subsequently putatively annotated in *Salinispora* strains by GNPS molecular networking and comparative MS^2^ analysis. Ketomemicins are assembled independently of ribosomal and NRPS pathways through an ATP-grasp peptide ligase-dependent biosynthetic system [52]. Ketomemicins C-418, C-432A, and C-432B (**81**–**83**) showed no antibacterial activity against *E. coli* MG1655 or *P. aeruginosa*. Meanwhile, **82** moderately inhibited the main proteases of SARS-CoV, SARS-CoV-2, and MERS-CoV, as well as cruzain, whereas **81** and **83** were inactive in these assays [52]. The *ktm* cluster contains six conserved genes, *ktmA–F*, encoding an aldolase, a PLP-dependent amino acid *C*-acyltransferase, a dehydratase, an ATP-grasp peptide ligase, an amidinotransferase, and a dehydrogenase. Related loci are broadly distributed across Actinomycetota. Ketomemicins C-390, C-482, and C-498 (**84**–**86**) were assigned from MS^2^ data, and their association with the *ktm* cluster has not been genetically validated in the corresponding strains [52].

## 7. Marine-Derived Rare Actinomycetes: *Saccharothrix*

### 7.1. Genus Overview and Biosynthetic Capacity

**Figure 10:**
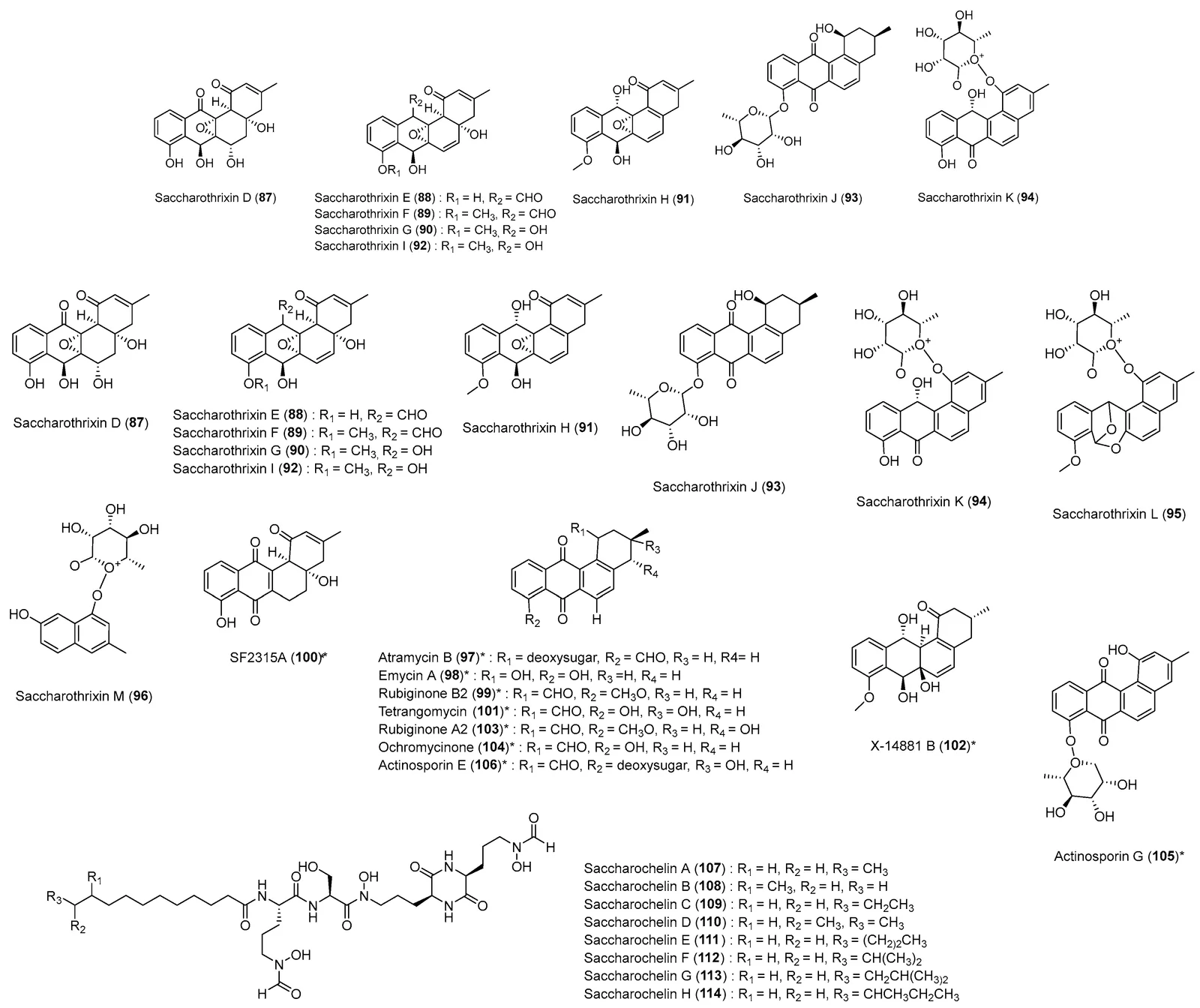
Structural overview of biosynthetic gene cluster (BGC)-linked metabolites from marine-derived *Saccharothrix* species. (*: previously reported compound).

*Saccharothrix* is a rare actinomycete genus distributed across terrestrial and marine environments [58]. Comparative genomic analysis of 25 high-quality *Saccharothrix* genomes identified 975 predicted BGCs grouped into 527 gene cluster families, many of which remain unlinked to characterized metabolites, indicating substantial cryptic biosynthetic potential [58]. Recent studies have combined genome mining, targeted gene deletion, and heterologous expression to link marine-derived *Saccharothrix* metabolites to their corresponding biosynthetic pathways [59,60]. The principal classes discussed below are type II PKS-derived angucyclines and angucyclinones and NRPS-derived amphiphilic siderophores. The corresponding chemical structures are presented in Figure 10, and the BGC-linked metabolites are summarized in Table 5.

### 7.2. Type II PKS Metabolites: Saccharothrixins D–M and Related Angucyclines

Saccharothrixins D–M (**87**–**96**), together with atramycin B (**97**), emycin A (**98**), rubiginone B2 (**99**), SF2315A (**100**), tetrangomycin (**101**), X-14881 B (**102**), rubiginone A2 (**103**), ochromycinone (**104**), and actinosporins G and E (**105** and **106**), constitute a structurally diverse family of type II PKS-derived angucyclines and angucyclinones isolated from the rare marine actinomycete *Saccharothrix* sp. D09. Saccharothrixins D–I (**87**–**92**) are highly oxygenated derivatives, whereas saccharothrixins J–M (**93**–**96**) are glycosylated analogs, highlighting the extensive oxidative and glycosylative tailoring of these aromatic polyketides [59]. Saccharothrixins F, G, and K (**89**, **90**, and **94**) showed antibacterial activity. **89** and **90** inhibited *Helicobacter pylori* 159 at 32 μg/mL, whereas **94** inhibited *H. pylori* G27, *H. pylori* 159, and *S. aureus* ATCC 25923 at 16 μg/mL. In addition, **89** also inhibited LPS-induced nitric oxide production in RAW264.7 macrophages, with an IC_50_ value of 28 μM [59].

Deletion of *sxnA1–A6* abolished production of the associated angucyclines, and heterologous expression of the intact *sxn* cluster reproduced the pathway products. These experiments functionally linked **87**–**106** to the *sxn* type II PKS pathway [59].

### 7.3. NRPS Metabolites: Saccharochelins

**Figure 11:**
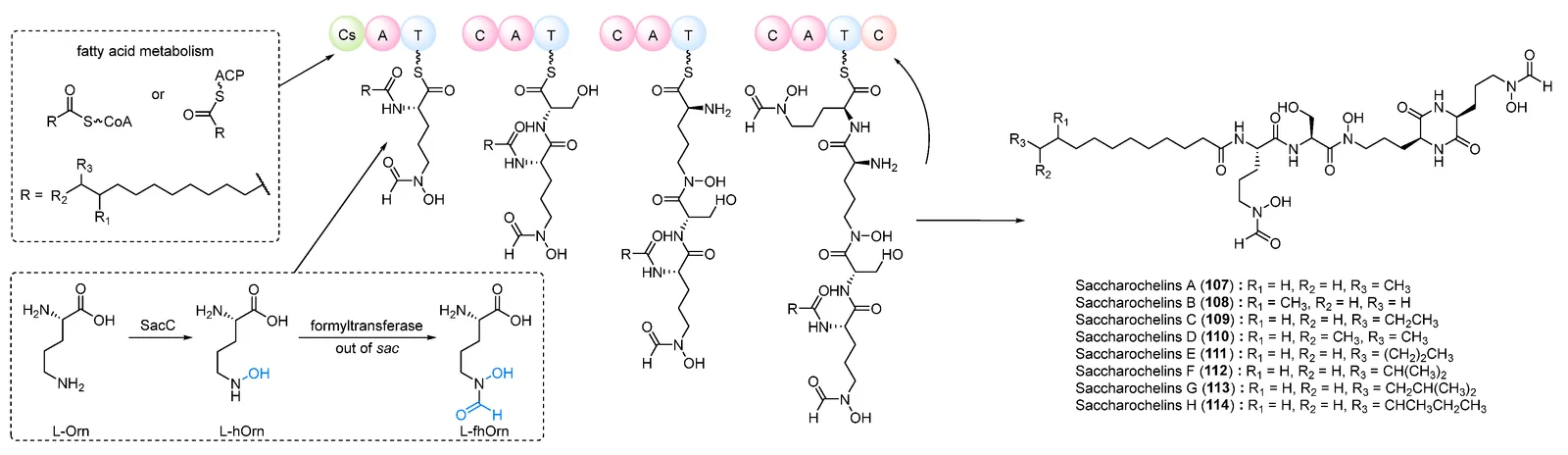
Proposed biosynthetic pathway of saccharochelins A–H (**107**–**114**) in *Saccharothrix* sp. D09 [60].

Saccharochelins A–H (**107**–**114**) are the first amphiphilic siderophores reported from *Saccharothrix*. They share a conserved tetrapeptide scaffold bearing linear or branched C12–C15 fatty acyl chains. Five tested congeners, **107**, **109**, **110**, **112**, and **113**, bound Fe(III) in the chrome azurol S assay [60]. **107**–**114** showed no antibacterial activity at concentrations up to 100 μM. Saccharochelins C, G, and H (**109**, **113**, and **114**) exhibited low-micromolar cytotoxicity against selected human cancer cell lines. Meanwhile, **114** was active against all four tested cell lines, with IC_50_ values of 3.3–5.9 μM; **113** showed the lowest individual IC_50_ value, 2.3 μM against HepG2 cells, whereas **109** inhibited A549, HCT-116, and HepG2 cells with IC_50_ values of 3.4–10 μM. The available structure–activity data suggest that the *N*-terminal fatty acyl chain influences cytotoxic potency [60]. Deletion of key NRPS modules within *sacE* abolished saccharochelin production, whereas heterologous expression of the intact *sac* cluster in *Streptomyces lividans* produced saccharochelins, functionally validating the pathway [60]. The proposed saccharochelin biosynthetic pathway is shown in Figure 11.

## 8. Marine-Derived Rare Actinomycetes: Minor Genera

### 8.1. Genus Overview and Biosynthetic Capacity

**Figure 12:**
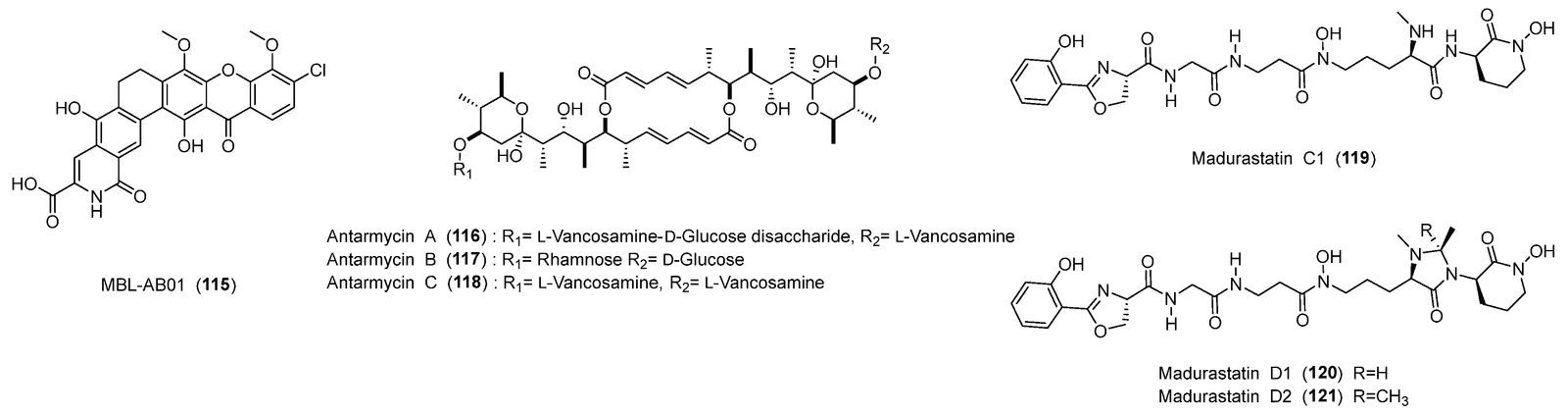
Structural overview of biosynthetic gene cluster (BGC)-linked metabolites from marine-derived minor rare actinomycete genera.

Less-explored marine-derived rare actinomycete genera produce structurally diverse secondary metabolites, including polycyclic xanthones, glycosylated macrodiolides, and phenolate hydroxamate siderophores [61,62,63]. *Pseudonocardia* is a rare actinomycete genus belonging to the family *Pseudonocardiaceae*. *Pseudonocardia* species have been isolated from diverse marine habitats, including marine sediments and marine sponges [64]. In the marine-derived strain *Pseudonocardia antarctica* SCSIO 07407, genome mining and genetic analyses identified the type I PKS ant cluster responsible for antarmycin biosynthesis [62]. *Actinomadura* belongs to the family *Thermomonosporaceae*, and marine-derived strains have yielded chemically diverse secondary metabolites [65]. Genome sequencing of *Actinomadura* sp. WMMA-1423 identified a putative *mad* NRPS locus associated with madurastatin biosynthesis [61]. *Actinoalloteichus* is a comparatively understudied actinomycete genus isolated from terrestrial and marine environments. Genome analysis of the marine isolate *Actinoalloteichus* sp. MP127-IG17 identified 30 predicted BGCs, including a putative type II PKS locus [63]. The structural classes discussed below include a polycyclic xanthone associated with a putative type II PKS BGC [63], type I PKS-derived glycosylated macrodiolides [62], and NRPS-derived phenolate–hydroxamate siderophores [61]. The corresponding chemical structures are presented in Figure 12, and the BGC assignments are summarized in Table 6.

### 8.2. Type II PKS Metabolites: MBL-AB01

MBL-AB01 (**115**) is a polycyclic xanthone antibiotic isolated from *Actinoalloteichus* sp. MP127-IG17. It is structurally related to xantholipin and lysolipin but contains an additional carboxyl group, suggesting oxidative modification of the polycyclic xanthone scaffold [63]. MBL-AB01 (**115**) showed potent antibacterial activity against Gram-positive pathogens, including methicillin-resistant *S. aureus* (MRSA), methicillin-susceptible *S. aureus* (MSSA), *M. luteus*, and vancomycin-resistant *E. faecium*. MIC values were 0.0078–0.032 μg/mL against *S. aureus*, 0.063 μg/mL against *M. luteus*, and 0.25–0.5 μg/mL against vancomycin-resistant *E. faecium*. No activity was detected against the tested Gram-negative bacteria. The EC_50_ values exceeded 20 μg/mL in five human cancer cell lines, whereas toxic effects toward IMR-90 human lung fibroblasts were observed at concentrations of ≥50 μg/mL. Serum reduced the antibacterial potency of 115 by 80- to 750-fold [63].

Genome analysis identified a putative 43-gene type II PKS locus for **115**, inferred from antiSMASH analysis and homology to the xantholipin and lysolipin biosynthetic gene clusters. A proposed pathway invokes C-22 hydroxylation and stepwise oxidation of the C-26 methyl group to a carboxylate to generate the characteristic hydroxylated and carboxylated polycyclic xanthone scaffold of **115**. The responsible enzymes and reaction order remain unassigned [63].

### 8.3. Type I PKS Metabolites: Antarmycins

Antarmycins A–C (**116**–**118**) are type I PKS-derived glycosylated macrodiolides; **116** was isolated from wild-type *Pseudonocardia antarctica* SCSIO 07407, whereas **117** and **118** were obtained from genetically engineered mutant strains. Moreover, **116** possesses a symmetric 16-membered macrodiolide scaffold bearing two l-vancosamine residues, one of which is further glucosylated. Meanwhile, **117** contains two α-l-rhamnose residues in place of the C-13-linked vancosamines, whereas **118** retains both vancosamines but lacks the glucose substituent [62]. In addition, **116** showed potent antibacterial activity against multidrug-resistant *E. faecium* and MRSA, with MIC values of 0.25–0.5 and 1–4 μg/mL, respectively. It was also active against *C. albicans* and *Cryptococcus neoformans*. Moreover, **117** was substantially less active against multidrug-resistant *E. faecium* and MRSA, with MIC values of 16–64 μg/mL, whereas **118** retained intermediate antibacterial activity, with MIC values of 0.125–16 μg/mL; **116** also showed rapid bactericidal activity and efficacy in intestinal and epicutaneous mouse infection models. Comparisons among **116**–**118** support an essential role for the l-vancosamine residues and indicate that additional glucosylation reduces antimicrobial potency. Mechanistic experiments further showed that **116** disrupts bacterial membranes through interactions with phosphatidylglycerol and phosphatidylethanolamine [62].

The plasmid-encoded *ant* cluster contains an eight-module type I PKS assembly line encoded by *antA1–antA5*, together with genes involved in l-vancosamine biosynthesis, glycosylation, transport, and self-resistance. Deletion of *antC* caused accumulation of the vancosamine-free aglycone, supporting its role in vancosamine transfer. The function of AntD was supported by deletion, complementation, and microsomal conversion of **118** into **116**. AntB7, an F420-dependent oxidoreductase, was genetically implicated in l-vancosamine biosynthesis and is proposed to catalyze the terminal C-4′ ketoreduction step [62].

### 8.4. NRPS Metabolites: (−)-Madurastatin C1, Madurastatins D1 and D2

(−)-Madurastatin C1 (**119**), madurastatins D1 and D2 (**120** and **121**) are NRPS-derived phenolate–hydroxamate siderophores isolated from *Actinomadura* sp. WMMA-1423. Specifically, **120** and **121** contain an unusual 4-imidazolidinone ring, whereas **119** possesses a linear α-amino amide moiety [61].

Moreover, **120** and **121** moderately inhibited *M. luteus*, with MIC values of 25.3 and 25.8 μM, respectively, while **119** was less active, with an MIC value of 108.1 μM. None of the new madurastatins inhibited MRSA at concentrations below 100 μM. All three compounds chelated Fe(III) in the chrome azurol S assay. In addition, **121** showed lower relative Fe(III)-binding efficiency, possibly because of its C-26 gem-dimethyl substitution [61].

**Figure 13:**
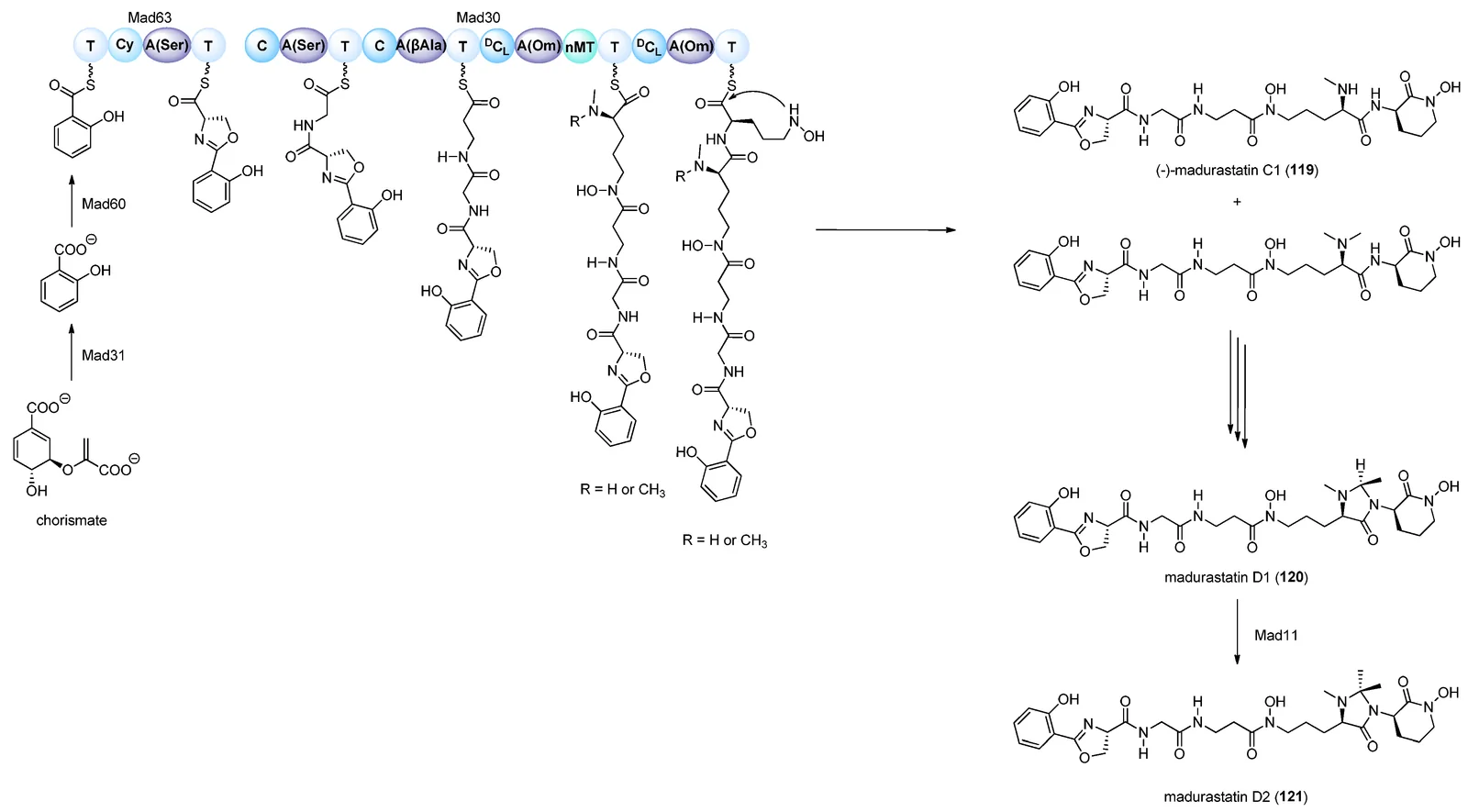
Proposed biosynthetic pathway of (−)-madurastatin C1 (119) and madurastatins D1 and D2 (120 and 121) in the sponge-derived marine *Actinomadura* sp. WMMA-1423 [61].

Genome analysis identified a putative *mad* biosynthetic locus comprising two co-localized NRPS clusters. Bioinformatic analysis assigned Mad30 and Mad63 as the core NRPS enzymes and proposed that Mad11-mediated methylation, followed by oxidation, contributes to formation of the 4-imidazolidinone ring in **120** and **121**. These functional assignments remain bioinformatically proposed and have not been genetically or biochemically validated [61]. The proposed biosynthetic pathway is shown in Figure 13.

## 9. Conclusions and Future Perspectives

Genome sequencing now reveals BGCs faster than their products can be isolated and assigned. For marine-derived rare actinomycetes, this imbalance remains a major limitation. Chemical access is biased toward strains that can be cultured reproducibly and toward genera for which genetic tools are available. Many predicted pathways remain silent or weakly expressed under laboratory conditions [10,19]. Even when a metabolite and a candidate BGC occur in the same strain, the assignment is often based on sequence similarity, domain organization, or metabolomic correlation rather than direct genetic evidence [16,17,18]. Genetic manipulation, cluster refactoring, and heterologous expression are still less routine for many rare actinomycetes than for established *Streptomyces* hosts, which limits access to low-abundance metabolites and complicates mechanistic studies [13,19]. Biological characterization is also uneven. Many compounds are evaluated in a limited set of *in vitro* assays, with little information on selectivity, molecular targets, resistance, or ecological function.

Progress will depend on closing these gaps experimentally. Comparative genomics can be paired with condition-resolved metabolomics so that BGC distribution, pathway expression, and product formation are evaluated in the same strain set [16,17,18]. Silent pathways can be addressed through cultivation changes, co-culture, nutrient or metal perturbation, pathway-specific regulatory engineering, and heterologous reconstruction [19,25,28]. Once a candidate cluster is linked to a metabolite, targeted deletion and complementation can define the genes required for production. Enzyme assays can then assign transformations that generate pathway branch points, unusual tailoring reactions, or stereochemical features. The mintaimycin study linked a predicted PKS–NRPS cluster to its products by heterologous expression and gene inactivation [24]. For kendomycin, cluster-level assignment and heterologous production [48] were followed by enzyme-level characterization of Kmy13 [50]. Computational prediction remains valuable for selecting experimentally tractable pathways, but it cannot substitute for pathway validation.

Much of the value of marine-derived rare actinomycetes lies in the biosynthetic chemistry that generates their final structures. Multi-locus biosynthesis, pathway crosstalk, module skipping, noncanonical substrate selection, oxidative coupling, and unusual halogenation or sulfur incorporation expose enzyme chemistry that can be utilized in pathway engineering and biocatalysis. These transformations warrant the same attention as the final metabolites. Biological characterization should likewise move beyond single-point activity measurements. Potency should be considered together with selectivity, mechanism of action, resistance liability, and the environmental context in which a metabolite is produced. A useful experimental sequence is to identify a BGC, establish its product, define the key enzymatic steps, and evaluate the resulting chemistry in biologically relevant systems. Applying this sequence consistently would convert a larger fraction of the predicted biosynthetic capacity of marine rare actinomycetes into experimentally defined metabolites and enzymes and provide a firmer basis for selecting compounds and pathways for downstream pharmacological or biosynthetic development.

## Figures and Tables

**Table 1:** Summary of biosynthetic gene cluster (BGC)-linked metabolites from marine-derived *Micromonospora* species.

Compound(s)	Structural Class	BGC Type/Key Genes
Mintaimycins A1, A2, A4–A6, and C (**1**–**6**)	PKS–NRPS hybrid peptide–polyketides	*min* PKS–NRPS hybrid BGC; validated by heterologous expression in *Streptomyces albus* J1074
Keyicin (**7**)	Bis-nitroglycosylated anthracycline	*kyc* type II PKS BGC; co-culture-induced activation by *Rhodococcus* sp. WMMA-185
Nenestatins B–I (**8**–**15**)	Benzo[*b*]fluorene atypical angucyclines	*nes* type II PKS BGC; Nes18 (dimerization) and Nes5 (α/β hydrolase)
Stealthins D–G (**16**–**19**)	Aminobenzo[*b*]fluorenes (fluostatin related)	*fls* BGC; FlsN3 (putative amidotransferase; function unresolved)
16-Demethylrifamycins (**20**–**23**)	Ansamycin/rifamycin-type polyketides	Cross-cluster: *pks3* (AHBA starter) + *M-rif* (rifamycin backbone)
IB-96212-type macrolides (**24**–**27**)	Spiroketal macrolides	Putative PKS/spirocyclase pathway (genome mining); not validated by knockout or heterologous expression
Neaumycin B (**28**)	Polycyclic macrolide	*neu* type I PKS BGC; genome-guided stereostructural assignment
Ecteinamines A–T (**29**–**48**)	Nonribosomal peptidic metallophores	*ect* NRPS BGC + menaquinone-derived *mqn* pathway
Dichloro-diketopiperazine (**49**)	Halogenated cyclic dipeptide	Co-isolated with **24**–**27**
Akazaoxime (**50**), A-76356 (**51**)	Enteromycin-class aldoxime	precursor feeding + total synthesis

**Table 2:** Summary of biosynthetic gene cluster (BGC)-linked metabolites from marine-derived *Nocardiopsis* species.

Compound(s)	Structural Class	BGC Type/Key Genes
Nocardiopsistins A–C (**52**–**54**)	Angucycline-type aromatic polyketides	Type II PKS BGC (KSα, KSβ/CLF, and ACP); isobutyryl-CoA starter + 9 malonyl-CoA
Nocardiopsistins D–F (**55**–**57**)	Brominated and sulfur-containing angucyclines	Same *ncd* type II PKS BGC; brominase NcdP (C-9 bromination)
Nocardioazine A (**58**)	Bridged prenylated diketopiperazine alkaloid	Proposed conversion from **59**; responsible enzyme and locus unresolved
Nocardioazine B (**59**)	Prenylated diketopiperazine Alkaloid	Split-locus CDPS pathway; NozA (*noz*) or NcdA (*ncd*) for precursor formation; NozR, NozPT, and NozMT (*noz2*) for tailoring

**Table 3:** Summary of biosynthetic gene cluster (BGC)-linked metabolites from marine-derived *Verrucosispora* species.

Compound(s)	Structural Class	BGC Type/Key Genes
Kendomycins B–D (**60**–**62**)	Carbacyclic ansa polyketides	*kmy* hybrid type I/III PKS BGC; Kmy13 (FAD monooxygenase); 3,5-DHBA starter
Verrucosins C–E (**63**–**65**)	Carbocyclic ansa polyketides	*vrs* hybrid type I/III PKS BGC (single locus, linear + cyclic)
Verrucosins A and B (**66** and **67**)	Linear polyketides	*vrs* hybrid type I/III PKS BGC
FW0622 (**68**)	Desferrioxamine- like Siderophore	NRPS-independent siderophore (NIS) pathway; DesA–DesD-like enzymes

**Table 4:** Summary of BGC-associated and putatively annotated metabolites reported from marine-derived *Salinispora* species.

Compound(s)	Structural Class	BGC Type/Key Genes
Lomaiviticins A, C–E (**69**–**72**)	Diazofluorene-containing aromatic polyketides	Type II PKS BGC (*lom* BGC) *lom58* knockout validation
Salinipyrone A and dehydrosalinipyrone A (**73** and **74**)	Truncated PKS byproducts	Type I PKS (*spr* BGC) *spr*7–*11*
Rosamicin A and its derivatives (**75**–**79**)	Macrolide polyketides	Type I PKS (*spr* BGC) *spr7*–*11*, P450 tailoring genes
18-Deoxo-5-deoxy-5-oxotylonolide (**80**)	Rosamicin-related aglycone	Type I PKS (*spr* BGC) *spr10* KR mutant product
Ketomemicins C-418, C-432A, and C-432B (**81**–**83**)	Ketomethylene-containing pseudopeptides	ATP-grasp peptide ligase (*ktm* BGC) *ktmA*–*F* (NRPS-independent)
Ketomemicins C-390, C-482, and C-498 (**84**–**86**)	Ketomethylene-containing pseudopeptides	*ktm* BGC (putative association not genetically validated) *ktmA*–*F* genes

**Table 5:** Summary of biosynthetic gene cluster (BGC)-linked metabolites from marine-derived *Saccharothrix* species.

Compound(s)	Structural Class	BGC Type/Key Genes
Saccharothrixins D–M (**87**–**96**)	Angucycline polyketides	Type II PKS (*sxn* BGC) *sxnA1–A6* deletion + heterologous expression
Atramycin B, Emycin A, Rubiginone B2, SF2315A, Tetrangomycin, X-14881 B, Rubiginone A2, Ochromycinone, Actinosporins G and E (**97**–**106**)	Angucycline polyketides	Type II PKS (*sxn* BGC) derived products
*Sac*charochelins A–H (**107**–**114**)	Siderophores	NRPS (*sac* BGC) *sac*E

**Table 6:** Summary of biosynthetic gene cluster (BGC)-linked metabolites from less-explored marine-derived rare actinomycete genera.

Compound(s)	Structural Class	BGC Type/Key Genes
MBL-AB01 (**115**)	Polycyclic xanthone	Putative type II PKS BGC
Antarmycins A–C (**116**–**118**)	Glycosylated macrodiolides	Type I PKS (ant BGC) *antA1–A5*, *antB1–B7*, *antC*, and *antD*
(−)-Madurastatin C1, Madurastatins D1 and D2 (**119**–**121**)	Siderophores	Putative NRPS *mad* locus (*mad* BGC) *mad30*, *mad63*, and *mad11*

## Data Availability

The original contributions presented in this study are included in the article. Further inquiries can be directed to the corresponding authors.
